# A Simple Mathematical Model for the Structural Description of Polyesters Based on Glycerol

**DOI:** 10.1021/acs.macromol.5c01193

**Published:** 2025-08-18

**Authors:** Giovanni B. Perin, Maria I. Felisberti

**Affiliations:** Institute of Chemistry, Universidade Estadual de Campinas (UNICAMP), P.O. BOX 6154, Campinas, São Paulo 13083-970, Brazil

## Abstract

Glycerol is a low-cost and biobased monomer widely used in the production of polyesters for applications such as surfactants and tissue engineering scaffolds, for example. However, the description of the structure of these polyesters remains challenging. This study presents a kinetic study of the polymerization of glycerol and dicarboxylic acids for the correlation of the ratio of the conversions of primary and secondary hydroxy groups of glycerol to their relative reactivity, the regioselectivity of the catalyst, and the occurrence of acyl migration. A mathematical model based on probability theory was used to estimate the molar fraction of the repetitive units of the polyesters, and key structural parameters such as the number-average degree of polymerization and the degree of branching as a function of conversion as well as the gel point were effectively described. These structural parameters were compiled into a theoretical structural map to provide a direct means of graphically understanding how the structure of the polyester changes as a function of hydroxy group conversion.

## Graphical abstract

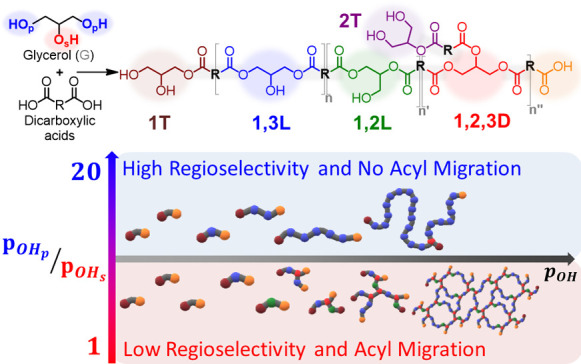

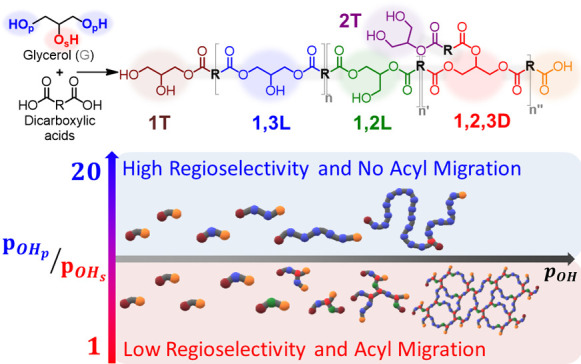

## 1 Introduction

Glycerol is the simplest polyol, composed of two primary and one secondary hydroxy groups.^1,2^ Its classification as “generally regarded as safe” by the United States Food and Drug Administration has led to its widespread use in pharmaceutical and healthcare products.^1,2^ Simultaneously, the increasing demand for polymeric materials has driven the development of new biobased alternatives to petroleum-derived materials.^3^ Among these, glycerol-based polyesters have attracted interest due to their biodegradation, biocompatibility, and low cost.^4^ Oligomers and branched polyesters from glycerol and dicarboxylic acids are amphiphilic because of the presence of pendent hydroxy groups along the polymer chain. These polyesters have been explored for various applications, including nanoparticles for drug delivery and biodegradable polymeric surfactants to solubilize hydrophobic molecules and stabilize emulsions and foams.^5–18^ As cross-linked elastomers, they are promising materials for tissue engineering due to their mechanical properties similar to biological tissues, as well as their shape-memory behavior, biocompatibility, and biodegradability.^19–27^ The presence of free hydroxy groups along the polyester chains further enables postfunctionalization with various molecules such as fatty acids,^6,8,9,28^ phosphate,^29,30^ amino acids,^16,31,32^ acetylcholine,^33^ norbornene,^34^ drugs,^17,35,36^ dyes,^15^ initiators of radical polymerization,^37^ anhydrides,^32^ and (meth)­acrylic groups,^15,20,38,39^ thereby expanding their range of applications.

Polycondensation between glycerol and a diacid follows the step-growth mechanism. In this mechanism, monomers react to form dimers, which can then react with other monomers to form trimers, and so on.^40^ Therefore, there is a slow increase in the polymer molar mass and the persistence of free monomers up to the gel point around *p*_OH_ ≈ 0.67–0.84.^41–50^ The traditional route to synthesize this kind of polyester involves the polycondensation of glycerol and dicarboxylic acids in bulk, under nitrogen flow or reduced pressure, and at temperatures of 120–150 °C.^1,3,19,46,51^ For the preparation of the elastomer, an additional curing step at reduced pressure and temperatures of 120–150 °C is also performed.^1,3,19,46,51^ Although this method is straightforward and cost-effective, it has limitations, including glycerol loss due to evaporation during polymerization and challenges in controlling the polyester structure.^1,3,19^ This approach typically produces randomly branched polyester oligomers comprising terminal (1T and 2T units), linear (1,3L and 1,2L units), and dendritic (1,2,3D) repetitive units (Figure 1a) and ultimately leads to cross-linked elastomers without reproducible properties.^1,3,46,47^

**Figure 1:**
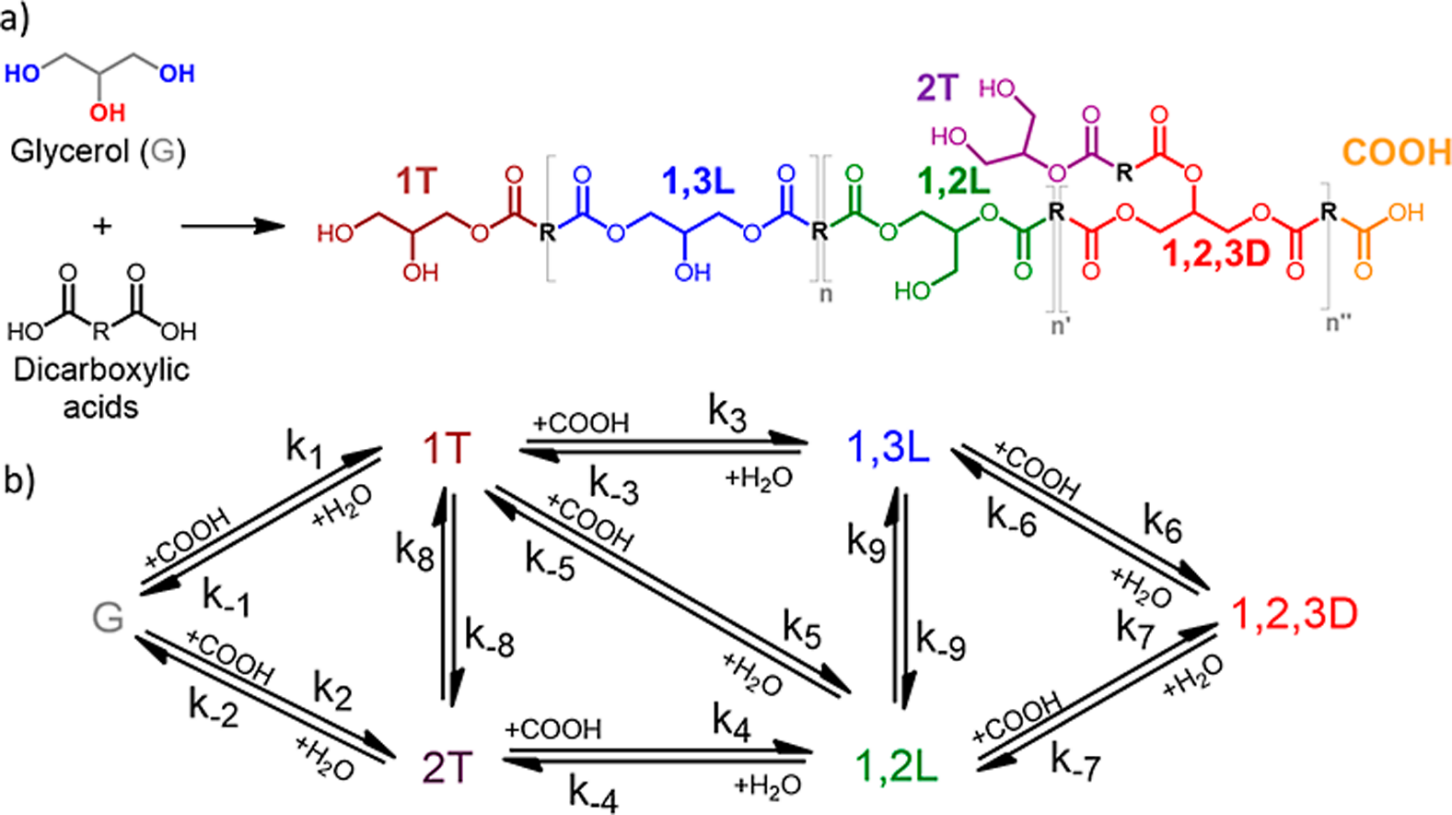
(a) Structure of the randomly branched polyester based on glycerol and dicarboxylic acids and (b) full mechanism of polymerization.

Strategies to avoid glycerol loss and to achieve fine structural control aiming at linear polyesters from glycerol have been explored in the literature using various strategies, including (i) the use of diarylborinic acids as the catalyst for the polycondensation of glycerol and diacyl chlorides in tetrahydrofuran in the presence of diisopropyl ethyl amine at 70 °C for 5 h;^31^ (ii) polymerization of the diglicidyl ester and dicarboxylic acids catalyzed by tetrabutylammonium bromide in DMF in the temperature range of 95–120 °C for 3–96 h, depending on the diacid structure^52^ and; (iii) the use of the enzyme *Candida antarctica* lipase B immobilized on the acrylic resin (CALB) as the catalyst for the polycondensation of glycerol and diacids/diesters in solution or bulk in the temperature range of 30–90 °C for up to 48 h.^6,47,53–61^ However, fine control of the structure of polyesters based on glycerol remains a challenge. Despite the difference in reactivity between the primary and secondary hydroxy groups of glycerol and the use of a regioselective catalyst, which potentially allows the synthesis of linear polyesters, the localization of the secondary hydroxy groups adjacent to the primary ones commonly results in the intramolecular acyl migration.^31,54,55,62^ Acyl migration reversibly converts esters of primary hydroxy groups into esters of secondary hydroxy groups toward the establishment of equilibrium at molar ratios of *x*_1T_/*x*_2T_ = 9 and *x*_1,3L_/*x*_1,2L_ = 1.5. This reaction is the main cause of the branching of these polyesters.^31,54,55,62^ Among the aforementioned approaches, the use of diarylborinic acid catalysts is the most effective in the synthesis of linear polyesters by suppressing acyl migration and reducing branching.^31^

In an attempt to predict the structure of polyesters based on glycerol, Li et al.^45^ used the probability theory to estimate the molar fractions of repetitive units of the poly­(glycerol sebacate) prepolymer and gel and compared them with experimental data obtained from carbon nuclear magnetic resonance (^13^C NMR) analysis. However, their model assumed equal reactivity between primary and secondary hydroxy groups, leading to significant discrepancies, once primary hydroxyls are 2–10 times more reactive than secondary ones under typical reaction conditions.^49,63–67^

Previously, we demonstrated that acyl migration occurs in parallel to regioselective esterification for the CALB-catalyzed polymerization of glycerol and diacids and is the main reaction responsible for polyester branching.^54,62^ Furthermore, at carboxylic acid conversion *p*_COOH_ > 0.90, the occurrence of acyl migration results in structurally similar polyesters.^54,55,62^

Herein, we provided a kinetic description of the polymerization of glycerol and dicarboxylic acids and used the ratio between the conversion of primary and secondary hydroxy groups (*p*_OH,p_/*p*_OH,s_) as a parameter to understand how the difference in the reactivity of primary and secondary hydroxy groups of glycerol (OH_p_ and OH_s_), the catalyst regioselectivity, and acyl migration influences the structure of the polyesters. The (*p*_OH,p_/*p*_OH,s_) ratio was incorporated in the probability theory for the construction of a mathematical model that allows the estimation of the molar fractions of the repetitive units that compose these polyesters.^65^ This model consistently describes the structure of polyesters based on glycerol and allows the estimation of the number-average degree of polymerization (Dp_*n*_ ) and degree of branching (DB) as a function of conversion as well as the gel point.

## 2 Methodology

All of the data used in this work were obtained from the literature, and the polymerization conditions are briefly described below.

The CALB-catalyzed polymerization of glycerol and diacids (adipic, suberic, sebacic, and dodecanedioic acids) in solution (acetone, acetonitrile, tetrahydrofuran, and tert-butanol) using molecular sieves and in bulk under nitrogen flow, in the temperature range from 40 to 80 °C, as well as the structural characterization of the polyesters were performed as reported elsewhere.^54,55,62^ The average molar fraction (*x*_*i*_) of residual glycerol and repetitive units (*i* = G, 1T, 2T, 1,3L, 1,2L, and 1,2,3D) that compose the polyester backbone in the reaction medium (Figure 1a) as well as the conversion of carboxylic acid and hydroxy groups (*p*_COOH_ and *p*_OH_) were calculated from ^1^H NMR data using the methodology described in a previous work.^54^ The conversion of the primary and secondary hydroxy groups of glycerol (*p*_OH,p_ and *p*_OH,s_) was calculated using the x_i_ values (available in previous works^54,55,62^ ) and eqs 1 and 2, respectively. The total hydroxy conversion (*p*_OH_) as a function of *p*_OH,p_, and *p*_OH,s_ is given by eq 3 and the relation between *p*_OH_ and *p*_COOH_ by eq 4.

1 $$p_{OH,p}=\frac{\left(x_{1T}+{2x}_{1,3L}+x_{1,2L}+{2x}_{1,2,3D}\right)}{2}$$

2 $$p_{OH,s}=x_{2T}+x_{1,2L}+x_{1,2,3D}$$

3 $$p_{OH}=\frac{\left(2p_{OH,p}+p_{OH,s}\right)}{3}=\frac{x_{1T}+x_{2T}+{2x}_{1,3L}+2x_{1,2L}+3x_{1,2,3D}}{3}$$

4 $$p_{COOH}=\frac{p_{OH}}{r}$$

where *r* = [COOH]_0_/[OH]_0_ is the molar ratio between the initial concentration of carboxylic acid and hydroxy groups.

Yang et al.^47^ reported the use of both CALB and dibutyltin oxide (DBTO) as the catalysts for the polymerization of glycerol and oleic diacid in bulk under reduced pressure at 80 and 150 °C, respectively, and used quantitative ^13^C NMR analysis to determine the polyester structure; Rao et al.^53^ reported the use of CALB as the catalyst for the polymerization of glycerol and diethyl suberate in diphenyl ether at 80 °C under reduced pressure and used ^1^H NMR analysis to determine the polyester structure; Pin et al.^68^ reported the noncatalyzed polymerization of glycerol and succinic acid in bulk at 150 °C using a Dean–Stark apparatus to remove water and used quantitative ^13^C NMR analysis to determine the polyester structure; and Zhang et al.^69^ reported the polymerization of glycerol and adipic acid in bulk using DBTO as a catalyst at 140 °C under nitrogen flow and used quantitative ^13^C NMR analysis to determine the polyester structure. In previous works,^47,53–55,61,68,69^ both ^13^C NMR and ^1^H NMR analyses were proven as suitable tools for the quantification of the molar fraction of the repetitive units of polyester chains. The data obtained from the above-cited works, as well as data after mathematical adequacy for our purposes, are presented in Tables S1–S4 (Supporting Information).

It is important to note that the mechanisms, approximations, and models described in this study for the polymerization of glycerol and dicarboxylic acids can also be applied to the polymerization of glycerol and dicarboxylic esters or diacyl chloride monomers. However, the results and discussion in this study focused exclusively on dicarboxylic acids for simplicity.

## 3 Results and Discussion

### 3.1 Kinetics of Polymerization of Glycerol and Dicarboxylic Acids and the *p*_OHp_/*p*_OHs_ Ratio

In general, the polymerization of glycerol and dicarboxylic acids produces branched polyesters consisting of terminal units 1T and 2T, linear units 1,3L and 1,2L, and dendritic units 1,2,3D (Figure 1a). This polymerization involves nine possible reversible steps, namely, seven esterification/hydrolysis and two acyl migration steps (Figure 1b). Consequently, the structure of the polyesters from glycerol is influenced by the relative reactivity of primary and secondary hydroxy groups of glycerol, the regioselectivity of the catalyst, and the occurrence or suppression of acyl migration. The detailed kinetic analysis considering the full mechanism is complex and typically requires solving a system of intricate equations.^70–73^ For a simplified analysis, we assumed: (i) primary and secondary hydroxy groups of glycerol are two distinct entities that have independent reactivity^48,65^ and (ii) the subproduct removal (water in the present study) was efficient enough for the esterification reaction to be considered irreversible.^74^ Under these assumptions, the complex nine-step reversible mechanism of glycerol and dicarboxylic acid polymerization (Figure 1b) can be deduced for two general cases of polymerization reported in the literature.

#### 3.1.1 Case I

Polymerization occurs under conditions of high regioselectivity for the esterification of primary hydroxy groups and with the suppression of acyl migration The polycondensation reported by Slavko & Taylor^31^ fits Case I. Linear polyesters prepared using diarylborinic acids as catalysts were composed mostly of 1,3L units (≅85%), only 3–15% of 1,2L units, and <1% of 1,2,3D. In this case, the full mechanism of polymerization (Figure 1b) can be rewritten as two sequential and irreversible esterifications of primary hydroxyls of glycerol, resulting in 1T and 1,3L units, Figure 2a. This mechanism can be further simplified from the point of view of functional groups, in this case, primary hydroxy groups (R_1_O_p_H), and carboxylic acid (R_2_COOH). This simplified mechanism considers the irreversible esterification of primary hydroxy groups present in glycerol and the 1T unit (R_1_O_p_H) and carboxylic acids (R_2_COOH) forming the ester of primary hydroxy groups (R_1_O_p_OCR_2_) present in the 1T and 1,3L units, Figure 2b, where *k*_p_ is the apparent rate constant of esterification of primary hydroxy groups.

**Figure 2:**
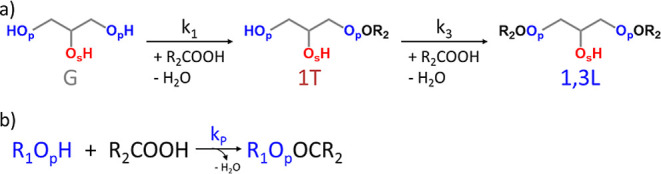
(a) Full mechanism and (b) simplified mechanism of the polymerization at high regioselectivity for the esterification of primary hydroxy groups and suppression of acyl migration (case I).

For the simplified mechanism of polymerization (Figure 2b), the rate law can be described as a second-order irreversible reaction. For glycerol/diacid equimolar ratio, [R_1_O_p_H]_0_ = [R_2_COOH]_0_, the rate law is given by eq 5, and the integral rate law (detailed deduction in the Supporting Information, “deduction of eq 6”) is given by eq 6.

5 $$\frac{d\left[R_{1}O_{p}H\right]}{dt}=-k_{p}\left[R_{1}O_{p}H\right]\cdot \left[R_{2}COOH\right]=-k_{p}{\left[R_{1}O_{p}H\right]}^{2}$$

6 $$\frac{1}{\left(1-p_{OHp}\right)}=1+{{\left[R_{1}O_{p}H\right]}_{0}k}_{p}t$$

where [R_1_O_p_H]_0_ is the initial concentration of primary hydroxy groups from glycerol, [R_1_O_p_H] is the concentration of primary hydroxy groups present in glycerol and repetitive units, [R_2_COOH] is the concentration of carboxylic acid, and *k*_p_ is the apparent rate constant of esterification of primary hydroxy groups.

#### 3.1.2 Case II

Polymerization occurs under conditions of regioselectivity for the esterification of primary hydroxy groups because primary hydroxy groups are intrinsically more reactive than secondary ones;^49,63–67^ however, acyl migration is present. The main example that fits case II is the polycondensation between glycerol and diacids/diesters using lipase as the catalyst and conducted in a solution of polar or apolar solvents as well as in bulk in the temperature range from 40 to 90 °C.^47,53–55^ For this, preferential esterification of primary hydroxy groups catalyzed by the lipase occurs at *p*_COOH_ ≤ 0.60–0.75.^62^ However, acyl migration becomes relevant at higher *p*_COOH_ values and results in polyesters with a structure characterized by the molar ratios *x*_1T_/*x*_2T_ ≈ 9 and *x*_1,3L_/*x*_1,2L_ ≈ 1.5.^54,62^ Two other polymerizations of glycerol and dicarboxylic acids reported in the literature also fit into case II: Pin et al.^68^ reported the noncatalyzed polymerization of glycerol and succinic acid at 150 °C using a Dean–Stark apparatus to remove water, and Zhang et al.^69^ polymerized glycerol and adipic acid in bulk using DBTO as the catalyst at 140 °C under a nitrogen flow to remove water vapor.

For case II, the occurrence of competitive second-order reactions (esterification of primary and secondary hydroxy groups and acyl migration) makes it challenging to deduce the integrated rate laws based on the full mechanism present in Figure 1b. However, a simpler analysis can be performed considering two ranges of carboxylic acid conversion, low (*p*_COOH_ ≤ 0.40) and high (*p*_COOH_ > 0.60–0.75, depending on the reaction conditions^62^), for which regioselective esterification and acyl migration are the main reactions, respectively.

At the beginning of the polymerization (*p*_COOH_ ≤ 0.40), the main reaction is the esterification of the primary and secondary hydroxy groups of glycerol and the formation of 1T and 2T units, as shown in Figure 3a. Acyl migration becomes relevant only at *p*_COOH_ ≥ 0.60–0.75.^62^ The mechanism shown in Figure 3a can be further simplified by assuming the occurrence of irreversible esterification of the primary and secondary hydroxy groups of glycerol (R_1_O_p_H and R_1_O_s_H, respectively) and carboxylic acids (R_2_COOH), and the formation of esters of primary and secondary hydroxy groups (R_1_O_p_OCR_2_ and R_1_O_s_OCR_2_, respectively) present in 1T or 2T units, respectively, according to Figure 3b, where *k*_p_ and *k*_s_ are the apparent rate constant of the esterification of primary and secondary hydroxy groups of glycerol, respectively.

**Figure 3:**
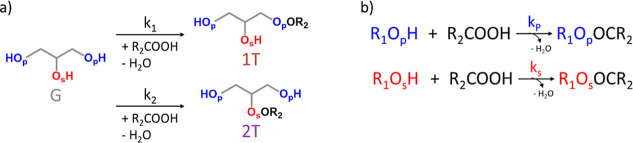
(a) Full mechanism and (b) simplified mechanism at low carboxylic acid conversion (*p*_COOH_ ≤ 0.40) that occur with regioselectivity for the esterification of primary hydroxy groups and absence of acyl migration (case II).

For the simplified mechanism of polymerization (Figure 3b), the rate laws can be described as second-order irreversible esterification of primary and secondary hydroxy groups, according to eqs 7 and 8, respectively.

7 $$\frac{d\left[R_{1}O_{p}H\right]}{dt}=-k_{p}\left[R_{1}O_{p}H\right]\cdot \left[R_{2}COOH\right]$$

8 $$\frac{d\left[R_{1}O_{s}H\right]}{dt}=-k_{s}\left[R_{1}O_{s}H\right]\cdot \left[R_{2}COOH\right]$$

Therefore, the relative reactivity of primary and secondary hydroxy groups of glycerol and/or catalyst regioselectivity can be estimated at the beginning of the polymerization (for *p*_COOH_ ≤ 0.40) by using eq 9. This equation is derived by dividing eq 7 by eq 8.^48^ The detailed deduction can be found in the Supporting Information—“deduction of eq 9”.

9 $$\frac{p_{OH,p}}{p_{OH,s}}\approx \frac{k_{p}}{{ak}_{s}}$$

where ***a*** is a constant that depends on the molar ratio *r* (eq 4) and the (*p*_OH,p_/*p*_OH,s_) ratio. For 0.5 < *r* < 1.0 and 2 < (*p*_OH,p_/*p*_OH,s_) < 20, the average value of the constant a is 1.3 ± 0.1 (Figures S1 and S2 and Table S5—Supporting Information).

According to a previous work,^62^ at a high carboxylic acid conversion (*p*_COOH_ ≥ 0.60–0.75, depending on the reaction conditions^62^), the reaction media is enriched with esters of primary hydroxy groups from the 1,3L unit (*p*_OH,p_ > 0.60–0.75) because *k*_p_ > *k*_s_, according to the literature.^49,63–67^ Furthermore, the relatively low concentration of free carboxylic acid slows the enzymatic esterification. These factors favor the conversion of esters of primary hydroxy groups from the 1,3L unit into esters of secondary hydroxy groups of the 1,2L unit by acyl migration, followed by further enzymatic esterification of the free primary hydroxy group of the 1,2L unit and formation of the 1,2,3D unit (Figure 4a). Therefore, the conversion of esters of primary hydroxy groups into esters of secondary hydroxy groups by acyl migration is the main reaction at *p*_COOH_ ≥ 0.60–0.75, Figure 4b. Moreover, at this stage of the polymerization, there is a net increase only in the conversion of secondary hydroxy groups from the 1,3L unit to the 1,2,3D unit, and a steady-state is achieved, in which the concentration of the esters of primary hydroxy groups is constant.^62^

**Figure 4:**
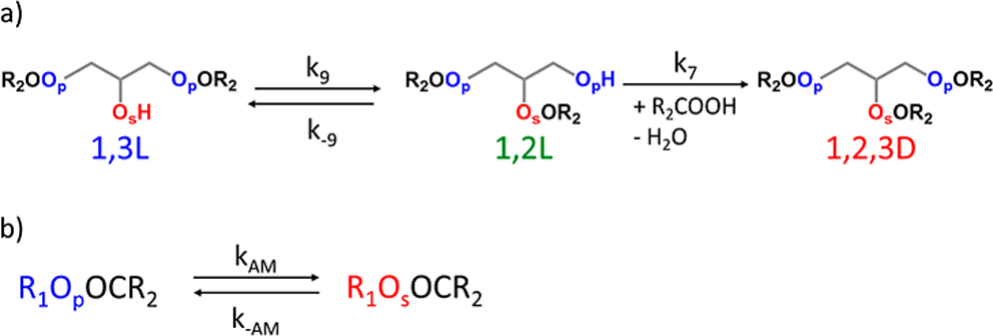
(a) Full mechanism and (b) simplified mechanism at high carboxylic acid conversion (*p*_COOH_ ≥ 0.60–0.75) that occur with regioselectivity for primary hydroxy groups and occurrence of acyl migration (case II).

For this simplified mechanism of polymerization (Figure 4b), the rate law can be described as a reversible conversion of esters of primary hydroxy groups into esters of secondary hydroxy groups according to eq 10 and the ratio between the direct and inverse acyl migration rate constants (*k*_AM_ and *k*_–AM_, respectively) can be obtained as the integrated rate law according to eq 11. Detailed deductions are provided in the Supporting Information—“deduction of eq 11“.

10 $$\frac{d\left[R_{1}O_{s}H\right]}{dt}\approx -k_{AM}\left[R_{1}O_{p}OCR_{2}\right]+k_{-AM}\left[R_{1}O_{s}OCR_{2}\right]$$

11 $$\frac{p_{OH,p}}{p_{OH,s}}\approx \frac{k_{-AM}}{{2k}_{AM}}$$

Equations 9 and 11 demonstrate that the relative reactivity of primary and secondary hydroxy groups and/or the regioselectivity of the catalyst as well as the occurrence of acyl migration can be evaluated by analyzing the (*p*_OH,p_/*p*_OH,s_) ratio at the beginning and late stages of the polymerization, respectively.

Figure 5 shows the plot of the (*p*_OH,p_/*p*_OH,s_) ratio as a function of *p*_COOH_ for various reaction conditions reported in the literature (data provided in Tables S1–S4, Supporting Information).

**Figure 5:**
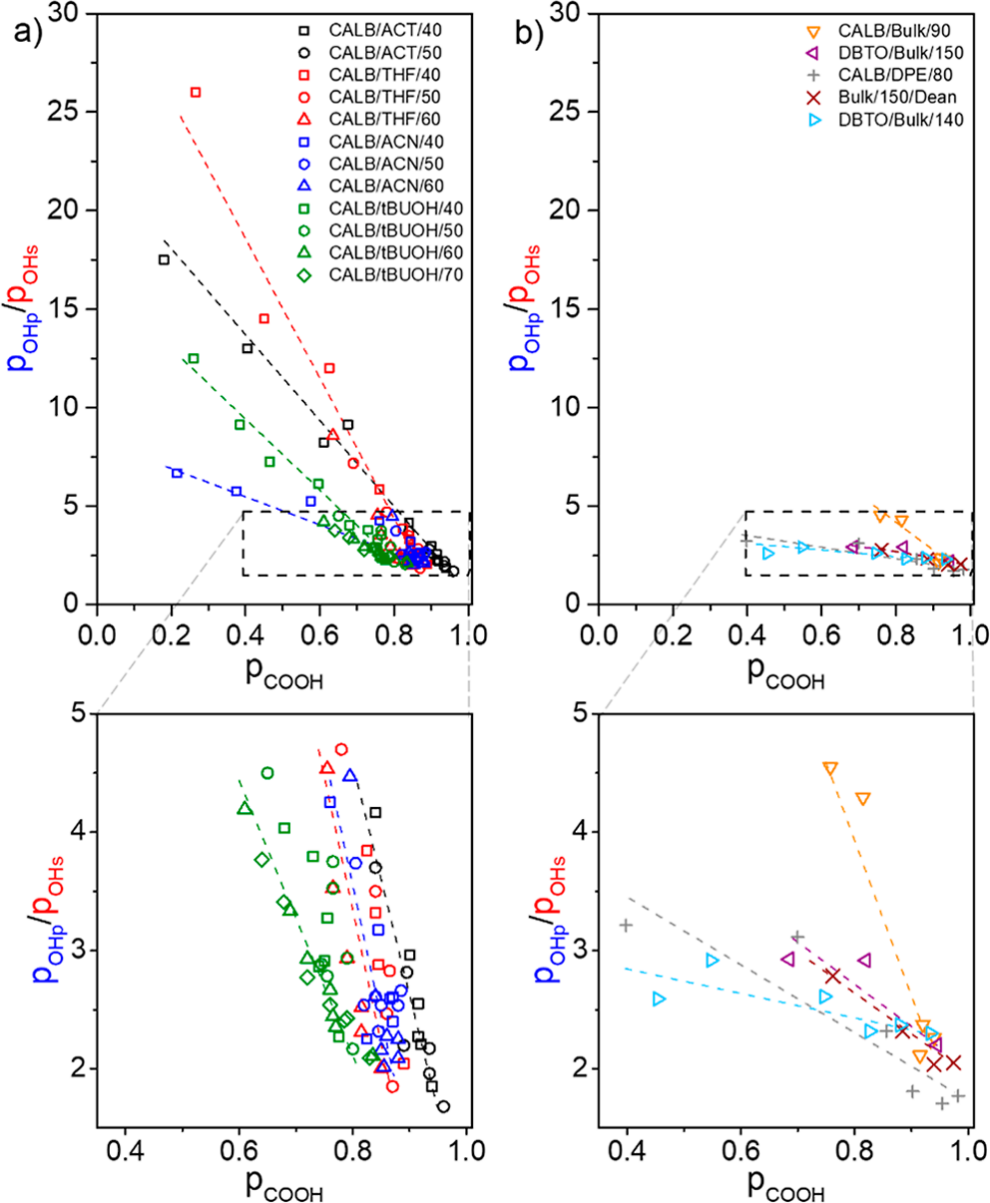
(*p*_OH,p_/*p*_OH,s_) ratio as a function of *p*_COOH_ for different reactions conditions: (a) CALB as the catalyst, for polymerization performed in acetone (ACT, black symbols), tetrahydrofuran (THF, red symbols), acetonitrile (ACN, blue symbols), or t-butanol (tBuOH, green symbols) as the solvent at 40 °C (□), 50 °C (○), 60 °C (△) or 70 °C (◇), for *r* = 0.67;^54^ (b) CALB (▽) and DBTO (◁) as the catalysts for reactions performed in bulk at 90 and 150 °C, respectively, for *r* = 0.67;^47^ CALB as the catalyst in diphenyl ether at 80 °C (+), for *r* = 0.67;^53^ uncatalyzed reaction at 150 °C (×), for *r* = 0.67;^68^ and DBTO as the catalyst for reactions in bulk at 140 °C (▷), for *r* = 0.50.^69^ A zoomed inset is provided below each figure for a better visualization of the data for *p*_COOH_ > 0.4.

Figure 5 shows a progressive decrease in the (*p*_OH,p_/*p*_OH,s_) ratio as the *p*_COOH_ increases. At low p_COOH_ values, the difference in the reactivity of primary and secondary hydroxy groups of glycerol and/or the regioselectivity of the catalyst is the main characteristic responsible for the relatively high value of the (*p*_OH,p_/*p*_OH,s_) ratio, according to eq 9. However, the occurrence of the acyl migration progressively decreases the value of the (*p*_OH,p_/*p*_OH,s_) ratio with increasing *p*_COOH_ until equilibrium is established at molar ratios *x*_1T_/*x*_2T_ = 9 and *x*_1,3L_/*x*_1,2L_ = 1.5, according to eq 11. Analysis of Figure 5 using eqs 9 and 11 indicates

12 limpCOOH→0(kpks)≈1.3(pOHppOHs)

13 limpCOOH→1(k−AMkAM)≈2(pOHppOHs)

At intermediate stages of the polymerization, the (*p*_OH,p_/*p*_OH,s_) ratio is a complex balance between the relative reactivity of primary and secondary hydroxy groups and/or the regioselectivity of the catalyst and acyl migration, and it depends mainly on the reaction conditions, as observed in Figure 5 and in agreement with a previous work.^62^ For example, the (*p*_OH,p_/*p*_OH,s_) vs *p*_COOH_ plots for the CALB-catalyzed reactions (Figure 5a) show that lipase is a regioselective catalyst because the conversion of primary hydroxy groups (*p*_OHp_) is higher than that of secondary ones (*p*_OHs_) at the early stage of the polymerization (*p*_OH,p_/*p*_OH,s_ > 5, therefore *k*_p_/*k*_s_ > 6.5). However, the occurrence of acyl migration progressively converts esters of primary hydroxy groups into esters of secondary hydroxy groups, reducing the (*p*_OH,p_/*p*_OH,s_) ratio. The values of the (*p*_OH,p_/*p*_OH,s_) ratio at the beginning of the reaction suggest that the regioselectivity of the CALB depends mainly on the solvent rather than on the temperature, and it is higher in acetone and tetrahydrofuran than in acetonitrile and *t*-butanol, as extensively discussed in a previous work.^62^ For the other reaction conditions reported in the literature, the data are only for *p*_COOH_ > 0.4, as shown in Figure 5b. In this case, acyl migration significantly contributes to the (*p*_OH,p_/*p*_OH,s_) values, which does not allow for a detailed analysis of the relative reactivity of primary and secondary hydroxy groups and the regioselectivity of the catalyst. However, the (*p*_OH,p_/*p*_OH,s_) values tend to approach 2 (and *k*_–AM_/*k*_AM_ tends to 4, in agreement with a previous work^62^) when *p*_COOH_ > 0.90 for all reaction conditions presented in Figure 5, resulting in polyesters with a similar distribution of the repetitive units 1T, 2T, 1,3L, 1,2L, and 1,2,3D.

### 3.3 Calculation of the Molar Fractions of the Repetitive Units

Brandner & Birkmeier^65^ proposed a mathematical model based on probability theory to estimate the molar fraction of mono-, di-, and triglycerides resulting from the esterification of glycerol with fatty acids. This model takes into account the different reactivities of primary and secondary hydroxy groups of glycerol as well as the occurrence of acyl migration by calculating the probability of primary and secondary hydroxy groups to be esterified as a function of the conversion. In the present work, the equations proposed by Brandner & Birkmeier^65^ were rewritten to calculate the molar fractions of residual glycerol (G) and repetitive units 1T, 2T, 1,3L, 1,2L, and 1,2,3D to express these parameters as a function of *p*_OHp_ and *p*_OHs_, as follows

14 $$x_{G}={\left(1-p_{OHp}\right)}^{2}\left(1-p_{OHs}\right)$$

15 $$x_{1T}=2\left(p_{OHp}\right)\left(1-p_{OHp}\right)\left(1-p_{OHs}\right)$$

16 $$x_{2T}={\left(1-p_{OHp}\right)}^{2}\left(p_{OHs}\right)$$

17 $$x_{1,3L}={\left(p_{OHp}\right)}^{2}\left(1-p_{OHs}\right)$$

18 $$x_{1,2L}=2\left(p_{OHp}\right)\left(1-p_{OHp}\right)\left(p_{OHs}\right)$$

19 $$x_{1,2,3D}={\left(p_{OHp}\right)}^{2}\left(p_{OHs}\right)$$

Equation 3 was also rearranged to express *p*_OHs_ and *p*_OHp_ as a function of the ratio (*p*_OH,p_/*p*_OH,s_) and *p*_OH_, according to eqs 20 and 21 (detailed deduction available in the Supporting Information—“deduction of eqs 20 and 21”). This strategy allows for the calculation of the molar fraction of the repetitive units (eqs 14–19) using the (*p*_OH,p_/*p*_OH,s_) ratio.

20 $$p_{OHs}=\frac{3}{\left[2\left(\frac{p_{OHp}}{p_{OHs}}\right)+1\right]}p_{OH}$$

21 $$p_{OHp}=\frac{3}{\left[2\left(\frac{p_{OHp}}{p_{OHs}}\right)+1\right]}\left(\frac{p_{OHp}}{p_{OHs}}\right)p_{OH}$$

The molar fractions of residual glycerol (G) and repetitive units 1T, 2T, 1,3L, 1,2L, and 1,2,3D determined experimentally by ^1^H NMR for polyesters obtained using CALB catalyst in acetone at 40 and 50 °C, and calculated using the spreadsheet S1 (Supporting Information), applying eqs 14–19 with the values of *p*_OHp_ and *p*_OHs_ derived from eqs 20 and 21, are presented in Figure 6a. The difference between the experimental values of the molar fractions (Δ*x*_*i*_) determined by ^1^H NMR and the calculated ones as a function of *p*_COOH_ is shown in Figure 6b. For other polymerization conditions presented in Figure 5, the experimental values of the molar fractions determined by NMR and the calculated ones, as well as the difference between these values, are provided in Figures S3–S13 (Supporting Information).

**Figure 6:**
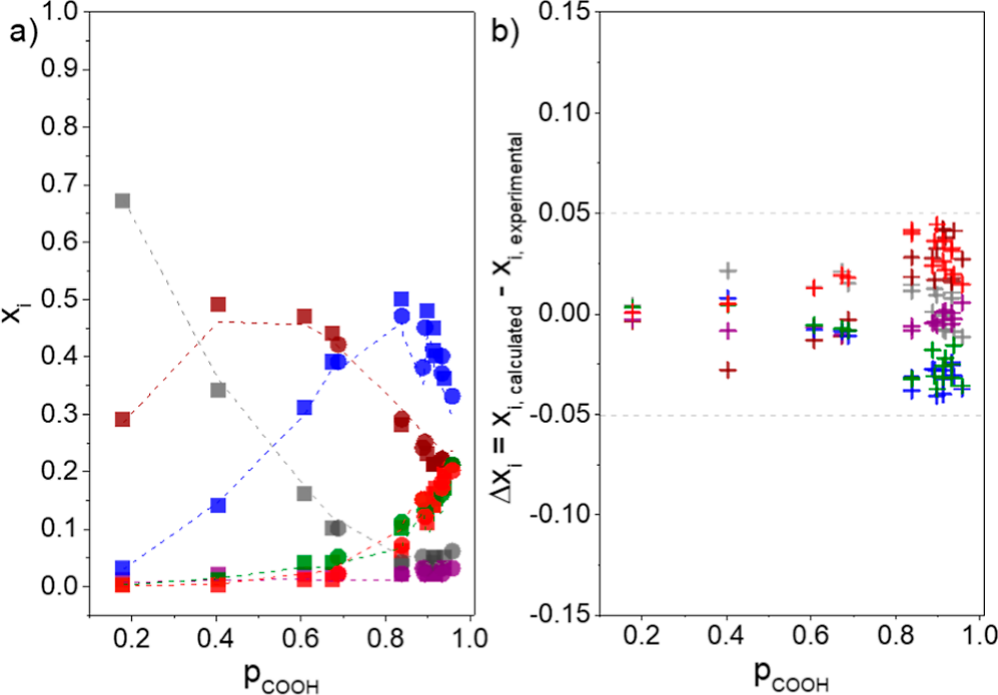
(a) Molar fraction (*x*_*i*_) of the residual glycerol (gray) and repetitive units 1T (wine), 2T (purple), 1,3L (blue), 1,2L (green), and 1,2,3D (red), determined by ^1^H NMR (*x*_*i*,experimental_—symbols), and calculated by eqs 14–19 (*x*_*i*,calculated_—lines), as a function of *p*_COOH_ for reactions performed using CALB as the catalyst in acetone at 40 °C (■) and 50 °C (●). b) Difference (Δ*x*_*i*_ = *x*_*i*,calculated_ – *x*_*i*,experimental_) between the values of the molar fraction determined experimentally by ^1^H NMR and the calculated ones using eqs 14–19.

Figure 6 and Figures S3–S13 (Supporting Information) demonstrate that eqs 14–19 are suitable for describing the molar fraction of repetitive units in polyesters based on glycerol with reasonable precision. Most of the difference (Δ*x*_*i*_) values fall within the range −0.05 ≤ Δ*x*_*i*_ ≤ 0.05, which is similar to the standard deviation values of around ±0.05 for the molar fractions of the repetitive units determined from ^1^H NMR data for replicates of CALB-catalyzed polymerizations of glycerol and dicarboxylic acids in solution (acetone and tetrahydrofuran) and in bulk.^55^ Equations 14–19 can also be applied to calculate the molar fractions of repetitive units of polyesters synthesized under different reaction conditions and molar ratio *r* = [COOH]_0_/[OH]_0_ (Figures S11–S13, Supporting Information), as well as for cross-linked elastomers,^45^ as shown in Table S6 (Supporting Information).

### 3.4 Theoretical Structural Mapping

The molar fraction of repetitive units, calculated using eqs 14–19 with the values of *p*_OHp_ and *p*_OHs_ calculated by eqs 20 and 21, allows further calculation of structural parameters such as DB (eq 22),^75^ number-average degree of polymerization (Dp_*n*_, eq 23),^54^ and the estimation of the gel point. According to Carothers,^50^ the gel point is defined as the hydroxy conversion p_OH_ at which a three-dimensional network with an infinitely high molar mass is formed. Therefore, the gel point could be defined as the p_OH_ value at which Dp_*n*_ increases steeply to values higher than 100. The spreadsheet S1 is available in the Supporting Information for the calculation of the molar fraction of the repetitive units, Dp_*n*_, and DB for variable (*p*_OH,p_/*p*_OH,s_) ratios and molar ratio *r* as a function of *p*_OH_.

22 $$DB=\frac{\left(2x_{1,2,3D}\right)}{\left(2x_{1,2,3D}+x_{1,3L}+x_{1,2L}\right)}\cdot 100$$

23 $${Dp}_{n}=\frac{\left\{\frac{1}{2}\left[x_{1T}+x_{2T}+\left(x_{ME}r\right)-x_{1,2,3D}\right]+\left(x_{DE}r\right)\right\}}{\left[\frac{1}{2}\left(x_{1T}+x_{2T}+\left(x_{ME}r\right)-x_{1,2,3D}\right)\right]}$$

where *x*_ME_ and *x*_DE_ are the molar fractions of mono- and diesters from the dicarboxylic acid monomer in the polymer chain calculated by eqs 24 and 25, respectively, according to the mathematical model proposed by Brandner & Birkmeier.^65^

24 $$x_{ME}=2\left(p_{COOH}\right)\left(1-p_{COOH}\right)$$

25 $$x_{DE}={\left(p_{COOH}\right)}^{2}$$

Figure 7 shows a “theoretical structural map” for polyesters based on glycerol, a plot of the (*p*_OH,p_/*p*_OH,s_) ratio as a function of p_OH_ for variable DB (Figure 7a) as well as Dp_*n*_ as a function of *p*_OH_ for variable (*p*_OH,p_/*p*_OH,s_) ratios (Figure 7b). Figure 7a,b is constructed using the spreadsheet S1 (data available in Tables S7–S11, Supporting Information). For Figure 7a, the (*p*_OH,p_/*p*_OH,s_) ratio was plotted as a function of *p*_OH_ at a given DB; the lever rule can be used to calculate DB values between two DB lines. The vertical black dotted lines represent the maximum p_OH_ at a given molar ratio *r* (i.e., *p*_OH_/*r* = *p*_COOH_ = 1.0); the light gray arrows mark the p_OH_ at the gel point for a specific molar ratio *r*; and in the light red area, *p*_OHp_ = 1.00 and a further increase in p_OH_ is only possible by changing the (*p*_OH,p_/*p*_OH,s_) ratio. For example, at (*p*_OH,p_/*p*_OH,s_) = 20 and *r* = 1.00, the maximum *p*_OH_ conversion is 0.68, at which *p*_OHp_ = 1.00 and *p*_OHs_ = 0.05 (estimated using eqs 21 and 20, respectively). In this case, *p*_OH_ values higher than 0.68 are achievable by increasing only *p*_OHs_, resulting in the decrease of the (*p*_OH,p_/*p*_OH,s_) ratio to values lower than 20, following the red line until (*p*_OH,p_/*p*_OH,s_) = 1 at *p*_OH_ = 1.00.

**Figure 7:**
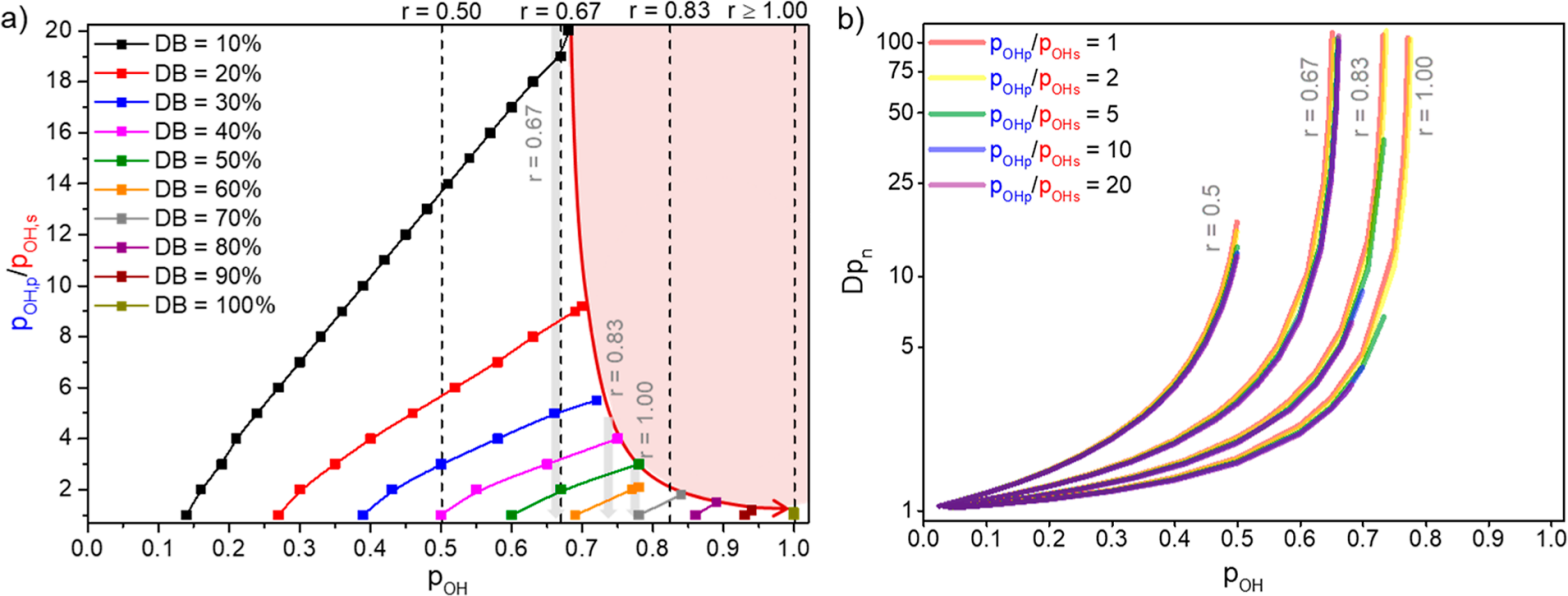
(a) “Structural map” of polyesters based on glycerol, a plot of the (*p*_OH,p_/*p*_OH,s_) ratio as a function of *p*_OH_ for variable DB and molar ratio *r*. (b) Number-average degree of polymerization (Dp_*n*_) as a function of *p*_OH_ for variable (*p*_OH,p_/*p*_OH,s_) ratios and *r* molar ratios.

The analysis of Figure 7a,b, supported by Spreadsheet S1, Supporting Information, provides a valuable tool for understanding how the structure of the polyesters based on glycerol changes as a function of *p*_OH_ for variable (*p*_OH,p_/*p*_OH,s_) and *r* ratios. In a theoretical scenario, it is possible to analyze how the polyester structure changes with increasing *p*_OH_ for a fixed *r* and (*p*_OH,p_/*p*_OH,s_) ratio in Figure 7a (horizontal tendency) and Figure 7b. For example, at (*p*_OH,p_/*p*_OH,s_) = 2.0 and *r* = 0.83, increasing *p*_OH_ from zero to 0.73 results in the formation of polyester with DB and Dp_*n*_ values up to 56% and 36, respectively (horizontal tendency in Figure 7a and yellow curve in Figure 7b). At *p*_OH_ = 0.74, a gel (gray arrow for *r* = 0.83, Figure 7a) is formed with a DB = 57%. Further, the DB of the gel increases to 67% as *p*_OH_ is increased to the maximum value of 0.83 (Figure 7a). On the other hand, it is also possible to analyze how the (*p*_OH,p_/*p*_OH,s_) ratio affects the polyester structure at a fixed *r* and *p*_OH_ in Figure 7a (vertical tendency) and Figure 7b. For example, at *p*_OH_ = 0.66 and *r* = 0.67, a gel with a DB = 56% is formed at (*p*_OH,p_/*p*_OH,s_) = 1, while a low-branched polyester with a DB = 9% and Dp_*n*_ = 63 is formed at (*p*_OH,p_/*p*_OH,s_) = 20 (vertical black dotted line, Figure 7a, and purple and red curves in Figure 7b). This tendency is in agreement with the fact that increasing the reactivity of primary hydroxy groups relative to secondary ones hinders the branching and cross-linking of the polyester.^47,48,69^

Figure 7b shows that Dp_*n*_ increases exponentially as a function of *p*_OH_, and Dp_*n*_ > 10 is generally achieved only at relatively high *p*_OH_/*r* values, consistent with the step-growth mechanism of polycondensation.^40,74^ For example, for *r* = 0.67, polyesters with Dp_*n*_ > 10 and low DB (≤10%, for *p*_OH,p_/*p*_OH,s_ ≥ 19, Figure 7a) can be formed because *p*_OH_/*r* ≥ 0.90 can be achieved through the selective esterification of primary hydroxy groups, i.e., increasing only *p*_OH,p_ (eq 3). For *r* > 0.67, polyesters with Dp_*n*_ > 10 are formed only for (*p*_OH,p_/*p*_OH,s_) < 10 (Figure 7b, green, yellow, and red lines for *r* = 0.83 and 1.0). In this case, polyesters with Dp_*n*_ < 10 are formed during the selective esterification of primary hydroxy groups up to *p*_OH,p_ = 1.0, and polyesters with Dp_*n*_ > 10 will be formed at the cost of the consumption of secondary hydroxy groups (and increase of *p*_OH,s_), which decreases the (*p*_OH,p_/*p*_OH,s_) ratio according to the dark red line in Figure 7a.

Regarding the gel point shown in Figure 7, it occurs at *p*_OH_ ≈ 0.77 for *r* = 1.00 and (*p*_OH,p_/*p*_OH,s_) ≤ 3; at *p*_OH_ ≈ 0.74 for *r* = 0.83 and (*p*_OH,p_/*p*_OH,s_) ≤ 4.3; and at *p*_OH_ ≈ 0.66 for *r* = 0.67 and (*p*_OH,p_/*p*_OH,s_) ≤ 20. The gel point does not occur for *r* = 0.5 (Figure 7b). In this case, the polyester with a maximum Dp_*n*_ of around 20 and DB = 40% could be formed. The *p*_OH_ values at the gel point are in agreement with both theoretical and experimental values reported in the literature, around *p*_OH_ ≈ 0.67–0.84, depending on the molar ratio *r*.^41–50^

## 4 Conclusion

The kinetic study of the polymerization of glycerol and dicarboxylic acids showed that the esterification of primary and secondary hydroxy groups of glycerol and the reversible acyl migration were the main reactions at the early and late stages of polymerization, respectively. Both the competitiveness between the esterification of primary and secondary hydroxy groups as well as the equilibrium of the acyl migration can be described using the (*p*_OH,p_/*p*_OH,s_) ratio. This strategy allowed us to correlate the influence of the relative reactivity of primary and secondary hydroxy groups, the regioselectivity of the catalyst, and the occurrence of acyl migration on the (*p*_OH,p_/*p*_OH,s_) ratio. The molar fractions of residual glycerol and repetitive units of the polyesters synthesized under different reaction conditions were estimated with high robustness by the incorporation of the (*p*_OH,p_/*p*_OH,s_) ratio into the mathematical model based on probability theory, and deviations from the experimental data are on the same order of magnitude as those observed between replicates of ^1^H NMR measurements . Structural parameters, including the number-average degree of polymerization and degree of branching as a function of the conversion of hydroxy groups, and the gel point, were estimated using the calculated molar fraction and compiled into a spreadsheet used to construct a theoretical structural map of polyesters based on glycerol. The theoretical structural map showed that the analysis of (*p*_OH,p_/*p*_OH,s_) as a function of the hydroxy group conversion provides a straightforward way to understand how the relative reactivity of primary and secondary hydroxy groups of glycerol, the regioselectivity of the catalyst, and the acyl migration affect the structure of these polyesters, influencing the degree of branching, number-average degree of polymerization, and gelation behavior. Therefore, this work pointed out that the (*p*_OH,p_/*p*_OH,s_) ratio and hydroxy conversion are the necessary and sufficient parameters for a full structural description of polyesters based on glycerol as the reaction progresses. The available spreadsheet will allow researchers to explore the model for other reaction conditions, in order to reach a universal understanding of how to tune the structure of these polyesters.

## Supplementary Material





## Notes

;Spreadsheet S1 used to calculate the structural parameters *x*_*i*_, *p*_OH_, *p*_COOH_, Dp_*n*,_ and DB as a function of (*p*_OHp_/*p*_OHs_) and molar ratio *r* and to calculate the data used for the construction of Figure 7 (XLSX)
Data from literature; deduction of eqs 6, 9, 11, 20, and 21; molar fractions of the repetitive units; and data for Figure 7 (PDF)
