# Supplementary material for: A Simple Mathematical Model for the Structural Description of Polyesters Based on Glycerol
Source: Macromolecules. 2025 Aug 18;58(16):8581–90. doi: 10.1021/acs.macromol.5c01193 (PMC12392719; doi:10.1021/acs.macromol.5c01193)
Supplement: Supplementary file 2 [file ma5c01193_si_002.pdf]

Supporting Information

A Simple Mathematical Model for the Structural Description of Polyesters Based on Glycerol

Giovanni B. Perin & Maria I. Felisberti*

Institute of Chemistry, Universidade Estadual de Campinas (UNICAMP), P.O. BOX 6154, 13083-970, Campinas - SP, Brazil

***Corresponding Author:** peringb@unicamp.br

SUMMARY

DATA FROM LITERATURE 1

DEDUCTION OF EQUATION 6 4

DEDUCTION OF EQUATION 9 5

DEDUCTION OF EQUATION 11 8

DEDUCTION OF EQUATIONS 20 AND 21 9

MOLAR FRACTIONS OF THE REPETITIVE UNITS 10

DATA FOR FIGURE 7 22

REFERENCES 27

Data from Literature

In this paper, a mathematical model was proposed aiming at the structural description of polyesters based on glycerol. This model is based on the theoretical calculation of the molar fraction (x_i) of each species i in the polymer backbones and residual glycerol (i = G, 1T, 2T, 1,3L, 1,2L, and 1,2,3D, as shown in Figure 1a) present in the reaction medium. To validate the model, we used our data and data from other authors. However, some data from the literature are related only to the composition of the polyester. For example, Yang *et al.*[1] and Pin *et al.*[2] reported in their works only the molar fraction (y_i) of the repetitive units (i = 1T, 2T, 1,3L, 1,2L, and 1,2,3D) in the polyester chains, neglecting the molar fraction of residual glycerol, and the conversion p_{OH} or p_{COOH}. For both works, we estimated graphically the molar fraction y_i. Because data reported by these authors are mostly for $p_{OH} \geq 0.45$, a condition for which $x_G \leq 0.15$, we used y_i values instead of x_i in **Equations 1 - 3**, as a reasonable approximation for the calculation of p_{OH}, $p_{OH,p}$, and $p_{OH,s}$ with deviations smaller than 10%.

On the other hand, Rao *et al.*,[3] reported both x_G and y_i (for i = 1T, 1,3L, 1,2L, and 1,2,3D). In this case, the values of x_i were calculated according to **Equation S1**.

$$x_i = (1 - x_G)y_i \qquad \textbf{(Equation S1)}$$

The values of x_i and/or y_i, as a function of time (t) reported by Yang *et al.*[1], Rao *et al.*[3], Pin *et al.*[2], and Zhang *et al.*[4], as well as the calculated values of p_{OH}, $p_{OH,p}$, $p_{OH,s}$ and ($p_{OH,p}$/$p_{OH,s}$) are presented in **Table S1 - S4**. For the CALB-catalyzed polycondensation of glycerol and sebacic acid in acetone, tetrahydrofuran, acetonitrile, and t-butanol at 40-70 °C, the x_i data are available in our previous works.[5,6]

Table S1: Molar fraction (y_i) of the repetitive units in the poly(oleic diacid-co-glycerol) chains synthesized using CALB/90 °C and DBTO/150 °C reported by Yang *et al.*[1] as well as the calculated values of p_{OH}, $p_{OH,p}$, $p_{OH,s}$, and the ratio ($p_{OH,p}/p_{OH,s}$).

t (h)	T (°C)	Catalyst	y_{1T}	$y_{1,3L}$	$y_{1,2L}$	$y_{1,2,3D}$	p_{OH}	$p_{OH,p}$	$p_{OH,s}$	($p_{OH,p}/p_{OH,s}$)
2	90	CALB	0.45	0.39	0.15	0.00	0.51	0.70	0.15	4.55
4	90	CALB	0.35	0.47	0.17	0.00	0.54	0.74	0.17	4.29
6	90	CALB	0.29	0.39	0.19	0.13	0.61	0.76	0.32	2.38
10	90	CALB	0.30	0.34	0.20	0.15	0.61	0.75	0.35	2.11
24	90	CALB	0.26	0.39	0.18	0.16	0.63	0.78	0.34	2.26
2	150	DBTO	0.59	0.19	0.20	0.00	0.46	0.6	0.20	2.93
4	150	DBTO	0.40	0.34	0.16	0.08	0.55	0.71	0.24	2.92
6	150	DBTO	0.30	0.36	0.18	0.17	0.63	0.76	0.35	2.20
8	150	DBTO	0.26	0.38	0.18	0.17	0.63	0.78	0.35	2.20

Table S2: Molar fraction (y_i) of the repetitive units in the poly(glycerol suberate) chains synthesized using CALB/diphenyl ether/80 °C and molar fraction of residual glycerol reported by Rao *et al.*[3] as well as the calculated values of x_i, p_{OH}, $p_{OH,p}$, $p_{OH,s}$, and the ratio ($p_{OH,p}/p_{OH,s}$).

t (h)	x_G	y_{1T}	$y_{1,3L}$	$y_{1,2L}$	$y_{1,2,3D}$	x_{1T}	$x_{1,3L}$	$x_{1,2L}$	$x_{1,2,3D}$	p_{OH}	$p_{OH,p}$	$p_{OH,s}$	($p_{OH,p}/p_{OH,s}$)
2	0.69	0.65	0.30	0.03	0.02	0.20	0.04	0.05	0.02	0.15	0.19	0.07	2.86
4	0.49	0.40	0.42	0.11	0.05	0.27	0.14	0.07	0.04	0.27	0.34	0.11	3.21
6	0.12	0.13	0.40	0.30	0.10	0.43	0.26	0.12	0.07	0.47	0.60	0.19	3.11
8	0.08	0.06	0.30	0.35	0.13	0.28	0.34	0.16	0.15	0.57	0.70	0.30	2.32
10	0.07	0.05	0.26	0.32	0.17	0.27	0.27	0.18	0.21	0.64	0.71	0.39	1.81
24	0.06	0.04	0.23	0.32	0.17	0.22	0.29	0.19	0.24	0.64	0.74	0.43	1.71
48	0.06	0.03	0.21	0.34	0.16	0.18	0.33	0.17	0.26	0.65	0.77	0.43	1.77

Table S3: Molar fraction (y_i) of the repetitive units in the poly(glycerol succinate) chains synthesized in bulk using DBTO/150 °C reported by Pin *et al.*[2] as well as the calculated values of p_{OH}, $p_{OH,p}$, $p_{OH,s}$, and the ratio ($p_{OH,p}/p_{OH,s}$).

t (h)	y_{1T}	y_{2T}	$y_{1,3L}$	$y_{1,2L}$	$y_{1,2,3D}$	p_{OH}	$p_{OH,p}$	$p_{OH,s}$	($p_{OH,p}/p_{OH,s}$)
1	0.47	0.07	0.31	0.11	0.05	0.51	0.64	0.23	2.78
3	0.31	0.04	0.38	0.16	0.11	0.59	0.72	0.31	2.32
5	0.25	0.04	0.38	0.18	0.15	0.63	0.75	0.37	2.04
7.18	0.23	0.02	0.40	0.19	0.18	0.65	0.78	0.38	2.05

Table S4: Molar fraction (x_i) of the residual glycerol and repetitive units in the poly(glycerol adipate) chains synthesized in bulk without a catalyst at 150 °C reported by Zhang *et al.*[4] as well as the calculated values of p_{OH}, $p_{OH,p}$, $p_{OH,s}$, and the ratio ($p_{OH,p}/p_{OH,s}$).

t (h)	x_G	x_{1T}	x_{2T}	$x_{1,3L}$	$x_{1,2L}$	$x_{1,2,3D}$	p_{OH}	$p_{OH,p}$	$p_{OH,s}$	($p_{OH,p}/p_{OH,s}$)
1.25	0.43	0.40	0.06	0.06	0.05	0.00	0.23	0.29	0.11	2.59
2	0.34	0.43	0.07	0.11	0.05	0.00	0.27	0.35	0.12	2.92
4	0.22	0.43	0.06	0.18	0.09	0.03	0.37	0.47	0.18	2.61
6	0.17	0.41	0.07	0.21	0.11	0.04	0.41	0.50	0.22	2.32
8.5	0.14	0.39	0.06	0.24	0.12	0.05	0.44	0.55	0.23	2.37
11.5	0.12	0.37	0.05	0.26	0.14	0.06	0.47	0.58	0.25	2.30

Deduction of Equation 6

In Case I, polycondensation is a simple second-order irreversible reaction according to **Equation 5**. At equimolar glycerol:diacid condition (r = 0.67), $[R_1O_pH]_0 = [R_2COOH]_0$, and the integral rate law can be described as a function of $[R_1O_pH]$ and $p_{OH,p}$, as follows.

$$\frac{d[R_1O_pH]}{dt} = -k_p[R_1O_pH]^2 \qquad \textbf{(Equation 5)}$$

Integrating and rearranging,

$$\frac{1}{[R_1O_pH]_0} - \frac{1}{[R_1O_pH]} = -k_p t \qquad \textbf{(Equation S2)}$$

Substituting, $[R_1O_pH] = (1-p_{OH,p})[R_1O_pH]_0$ and rearranging,

$$\frac{1}{(1-p_{OH,p})} = 1 + [R_1O_pH]_0 k_p t \qquad \textbf{(Equation 6)}$$

where $[R_1O_pH]_0 = 2[\text{Glycerol}]_0$

Deduction of Equation 9

For Case II, the relative reactivity of primary and secondary hydroxy groups of glycerol can be estimated at the beginning of the polymerization by the k_p/k_s ratio, by dividing **Equation 7** by **Equation 8**, as detailed as follows.

$$\frac{\frac{d[R_1O_pH]}{dt}}{\frac{d[R_1O_sH]}{dt}} = \frac{k_p[R_1O_pH]}{k_s[R_1O_sH]} \qquad \textbf{(Equation S3)}$$

Integrating and rearranging:

$$\left[ln\left(\frac{[R_1O_pH]}{[R_1O_pH]_0}\right)\right] = \left(\frac{k_p}{k_s}\right)\left[ln\left(\frac{[R_1O_sH]}{[R_1O_sH]_0}\right)\right] \qquad \textbf{(Equation S4)}$$

Substituting $[R_1O_pH]/[R_1O_pH]_0 = (1\text{-}p_{OH,p})$ and $[R_1O_sH]/[R_1O_sH]_0 = (1\text{-}p_{OH,s})$ in **Equation S4**, and rearranging it, the relative reactivity of primary and secondary hydroxy groups and/or regioselectivity of the catalyst can be estimated as k_p/k_s ratio by **Equation S5**.

$$\frac{\ln(1- p_{OH,p})}{\ln(1-p_{OH,s})} = \left(\frac{k_p}{k_s}\right) \qquad \textbf{(Equation S5)}$$

A further approximation can be performed for $p_{COOH} < 0.4$, $0.5 \leq r \leq 1.0$ and $2 \leq (p_{OH,p}/p_{OH,s}) \leq 20$, according to **Equation S6**; the ratio k_p/k_s can be estimated as the slope of the curves $\ln(1\text{-}\ p_{OH,p})$ *vs.* $\ln(1\text{-}\ p_{OH,s})$ or as a product of a constant **a** and the slope of the curve $p_{OH,p}$ *vs.* $p_{OH,s}$ obtained from data calculated using the **Spreadsheet S1** and present in **Figures S1** and **S2**, and **Table S5**.

$$\left(\frac{k_p}{k_s}\right) = \frac{\ln(1- p_{OH,p})}{\ln(1-p_{OH,s})} \approx \boldsymbol{a}\left(\frac{p_{OH,p}}{p_{OH,s}}\right) \qquad \textbf{(Equation S6)}$$

For $0.5 < r < 1.0$ and $2 < (p_{OH,p}/p_{OH,s}) < 20$, the average value of the constant **a** is 1.3 ± 0.1 (**Table S5**).

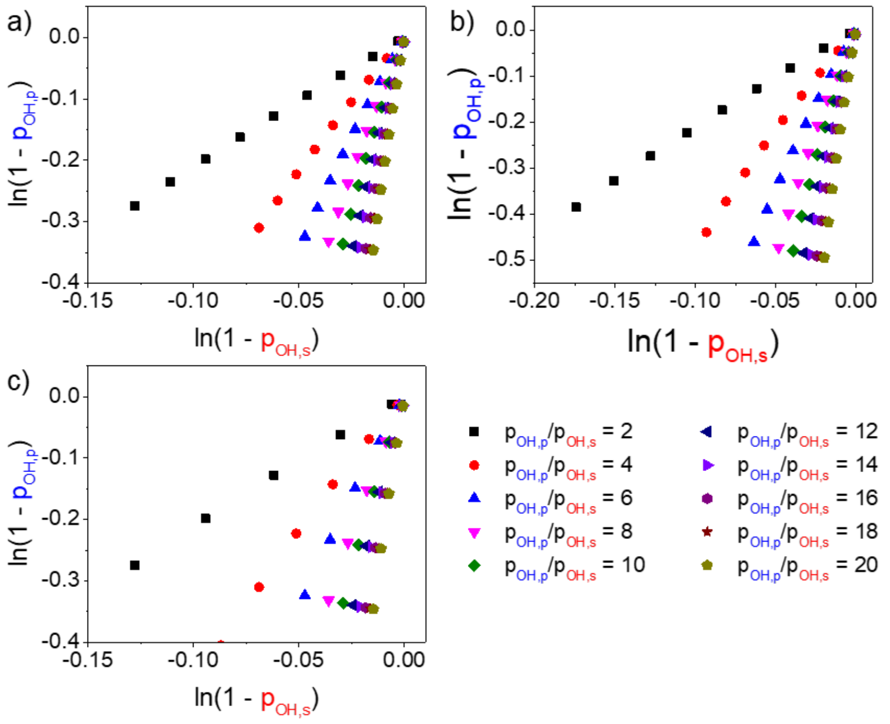

Figure S1: Plots of ln(1-$p_{OH,p}$) as a function of ln(1-$p_{OH,s}$) for $2 \leq (p_{OH,p}/p_{OH,s}) \leq 20$, $p_{COOH} < 0.40$, and variable molar ratio r: **a)** 0.5, **b)** 0.67, and **c)** 1.0.

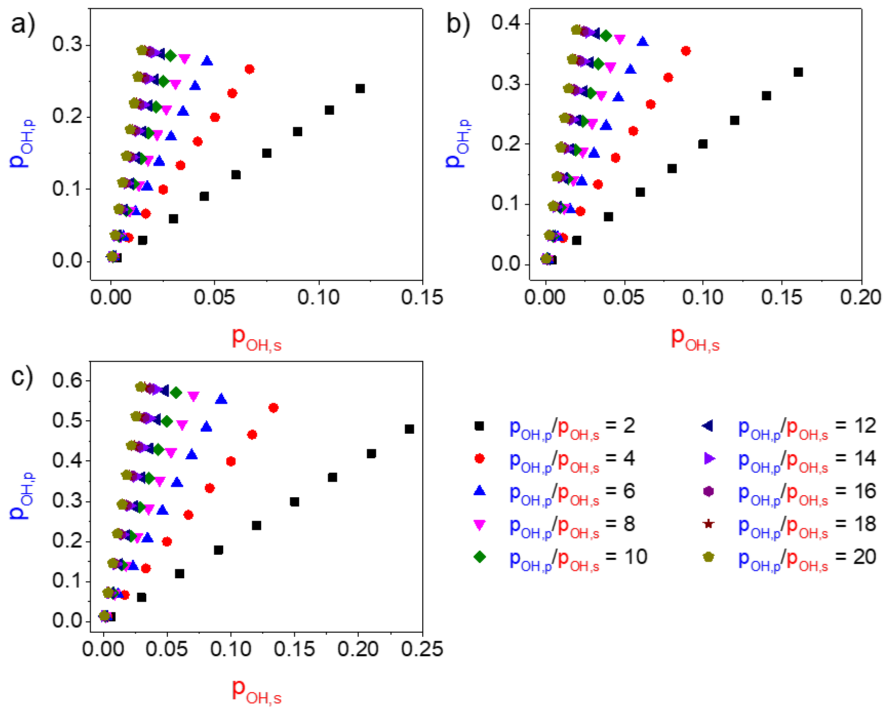

Figure S2: Plots of $p_{OH,p}$ as a function of $p_{OH,s}$ for $2 \leq (p_{OH,p}/p_{OH,s}) \leq 20$, $p_{COOH} < 0.40$, and variable molar ratio r: **a)** 0.5, **b)** 0.67 and, **c)** 1.0.

Table S5: k_p/k_s and $p_{OH,p}/p_{OH,s}$, the slope of the linear regression of the curves in **Figure S1** and **S2**, respectively, and constant **a**, according to **Equation S6**, for $p_{COOH} < 0.4$, $2 \leq (p_{OH,p}/p_{OH,s}) \leq 20$, and $0.5 < r < 1.0$.

	r = 0.5		r = 0.67		r = 1.0	
$\mathbf{p_{OH,p}/p_{OH,s}}$	k_p/k_s	**a**	k_p/k_s	**a**	k_p/k_s	**a**
2	2.1	1.1	2.2	1.1	2.4	1.2
4	4.5	1.1	4.7	1.2	5.3	1.3
6	6.9	1.1	7.2	1.2	8.3	1.4
8	9.2	1.2	9.8	1.2	11.3	1.4
10	11.6	1.2	12.3	1.2	14.3	1.4
12	14.0	1.2	14.9	1.2	17.3	1.4
14	16.4	1.2	17.4	1.2	20.3	1.4
16	18.7	1.2	20.0	1.2	23.3	1.5
18	21.1	1.2	22.5	1.3	26.3	1.5
20	23.5	1.2	25.1	1.3	29.3	1.5
Average ± Standard Deviation = 1.3 ± 0.1						

Deduction of Equation 11

The esterification slows down at the later stage of the polymerization, $p_{COOH} > 0.60$ - 0.75, and acyl migration becomes the main reaction (Case II). In this case, **Equation 10** can be used for the estimation of the acyl migration rate constants.

$$\frac{d[R_1O_sH]}{dt} \approx -k_{AM}[R_1O_p\mathrm{OC}R_2] + k_{-AM}[R_1O_s\mathrm{OC}R_2] \quad \textbf{(Equation 10)}$$

According to previous work,[6] at $p_{COOH} \geq 0.60$ - 0.75, there is an enrichment of the reaction medium with ester of primary hydroxy groups because $k_p > k_s$. Therefore, the acyl migration occurs mainly towards the conversion of 1,3L units into 1,2L units (**Figure 4**).[6] From this point $p_{OH,p} \approx$ constant, indicating a steady-state, for which the ester of primary hydroxy groups are an intermediate species and $d[R_1O_pH]/dt \approx 0$.[6] Substituting $[R_1O_pOCR_2] = [R_1O_pH]_0 p_{OH,p}$ and $[R_1O_sOCR_2] = [R_1O_sH]_0 - [R_1O_sH]$ in **Equation 10**, and integrating, k_{AM} and k_{-AM} can be estimated by an exponential fit of **Equation S7**, and $2k_{AM}/k_{-AM}$ and k_{-AM} as the fitting parameters, as reported in our previous work.[6]

$$\frac{p_{OH,s}}{p_{OH,p}} = \left(\frac{2k_{AM}}{k_{-AM}}\right)\left(1 - e^{-k_{-AM}t}\right) \quad \textbf{(Equation S7)}$$

The values of k_{-AM} reported in the literature[6] vary in the range from 0.1 to 0.59, the exponential term is around zero for $t \geq 24$ h, a reaction time that generally the $p_{COOH} >$ 0.75, and the acyl migration predominates. In this condition, the exponential term in **Equation S7** tends to zero, and **Equation S7** can be simplified to **Equation 11**

$$\frac{p_{OH,s}}{p_{OH,p}} \approx \left(\frac{2k_{AM}}{k_{-AM}}\right) \quad \textbf{(Equation 11)}$$

Deduction of Equations 20 and 21

The relationship between p_{OH} to $p_{OH,p}$ and $p_{OH,s}$ is given by **Equation 3**.

$$p_{OH} = \frac{(2p_{OH,p}+p_{OH,s})}{3} \qquad \textbf{(Equation 3)}$$

Dividing both sides by $p_{OH,s}$, and rearranging **Equation 3**:

$$\frac{p_{OH}}{p_{OH,s}} = \frac{\left[2\left(\frac{p_{OH,p}}{p_{OH,s}}\right)+1\right]}{3} \qquad \textbf{(Equation S8)}$$

Multiplying both sides by $p_{OH,p}$, and rearranging:

$$p_{OH}\left(\frac{p_{OH,p}}{p_{OH,s}}\right) = \frac{\left[2\left(\frac{p_{OH,p}}{p_{OH,s}}\right)+1\right]}{3}p_{OH,p} \qquad \textbf{(Equation S9)}$$

Equation S9 can be further rearranged in **Equation 20** and **Equation 21**.

$$p_{OH,s} = \frac{3}{\left[2\left(\frac{p_{OH,p}}{p_{OH,s}}\right)+1\right]}p_{OH} \qquad \textbf{(Equation 20)}$$

$$p_{OH,p} = \frac{3}{\left[2\left(\frac{p_{OH,p}}{p_{OH,s}}\right)+1\right]}\left(\frac{p_{OH,p}}{p_{OH,s}}\right)p_{OH} \qquad \textbf{(Equation 21)}$$

Molar Fractions of the Repetitive Units

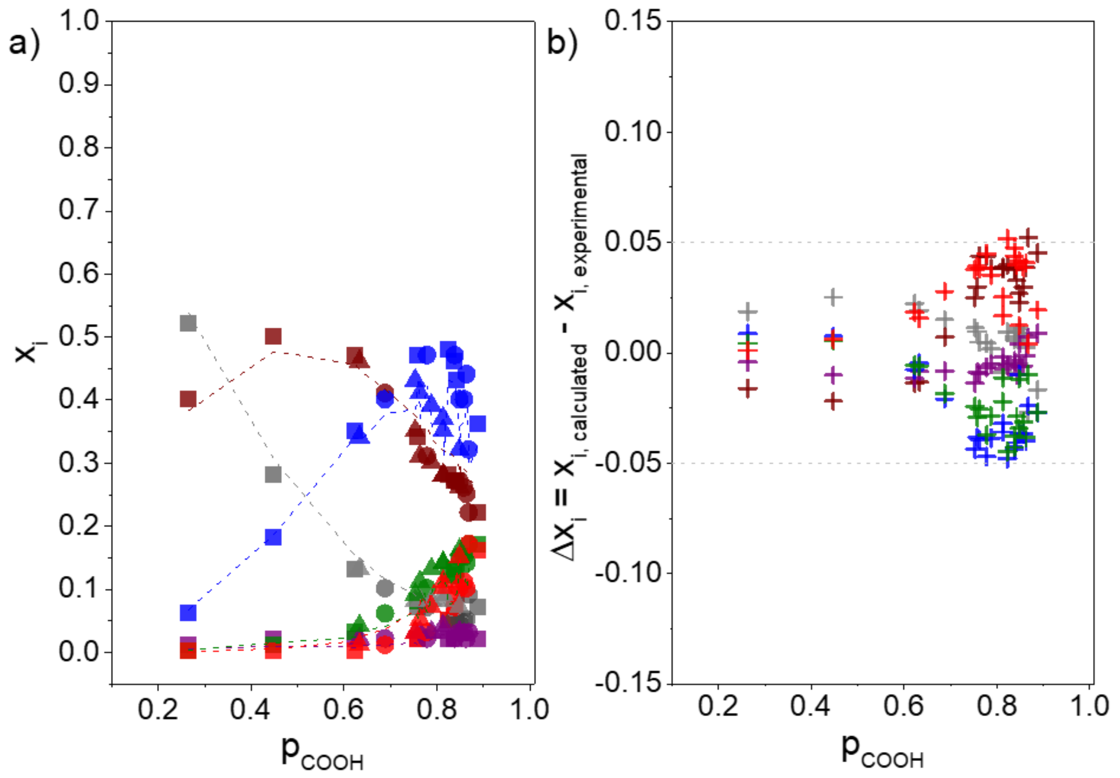

Figure S3: a) Molar fraction (x_i) of the residual glycerol (gray) and the repetitive units 1T (wine), 2T (purple), 1,3L (blue), 1,2L (green), and 1,2,3D (red), determined by ^{1}H NMR ($x_{i,\ experimental}$ - symbols), and calculated by **Equations 14 - 19** ($x_{i,\ calculated}$ - dashed lines), as a function of p_{COOH} (r = 0.67) for reactions performed using **CALB as the catalyst in tetrahydrofuran** at 40 °C (■), 50 °C (●), and 60 °C (▲).[5] **b)** Difference ($\Delta x_i = x_{i,\ calculated} - x_{i,\ experimental}$) between the molar fraction values determined experimentally by ^{1}H NMR and the calculated ones.

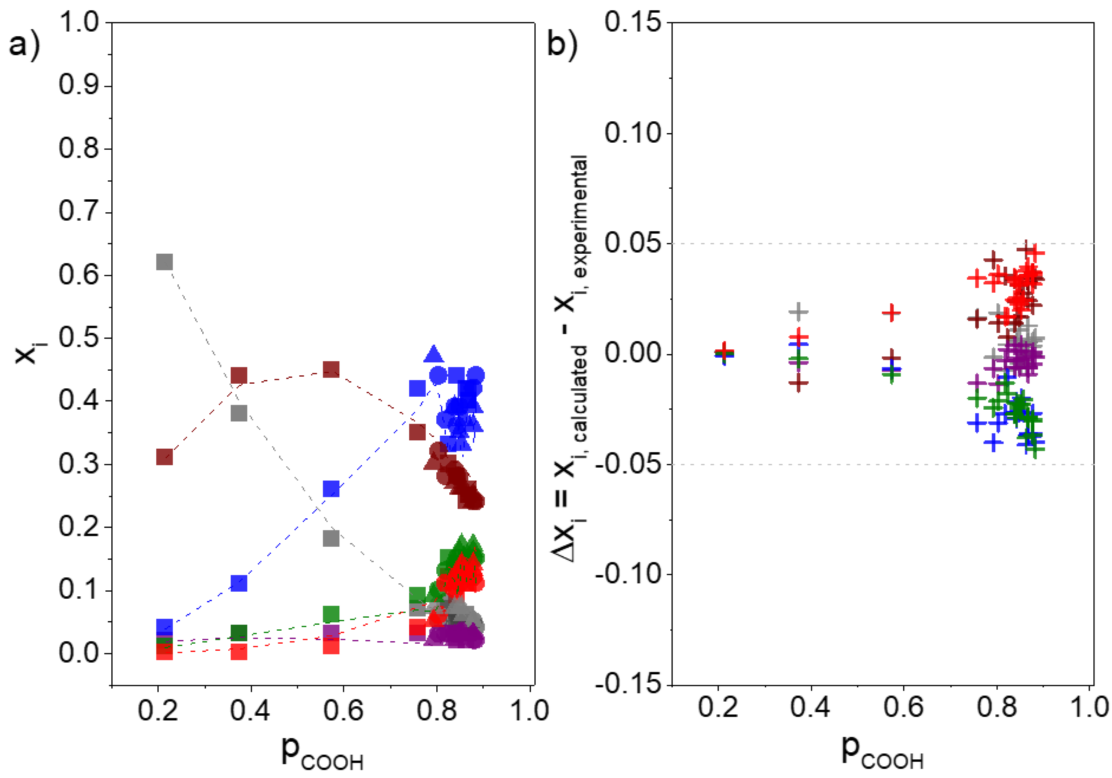

Figure S4: a) Molar fraction (x_i) of the residual glycerol (gray) and the repetitive units 1T (wine), 2T (purple), 1,3L (blue), 1,2L (green), and 1,2,3D (red), determined by ^{1}H NMR ($x_{i,\,experimental}$ - symbols), and calculated by **Equations 14 - 19** ($x_{i,\,calculated}$ - dashed lines), as a function of p_{COOH} (r = 0.67) for reactions performed using **CALB as catalyst in acetonitrile** at 40 °C (■), 50 °C (●), and 60 °C (▲).[5] **b)** Difference ($\Delta x_i = x_{i,\,calculated} - x_{i,\,experimental}$) between the molar fraction values determined experimentally by ^{1}H NMR and the calculated ones.

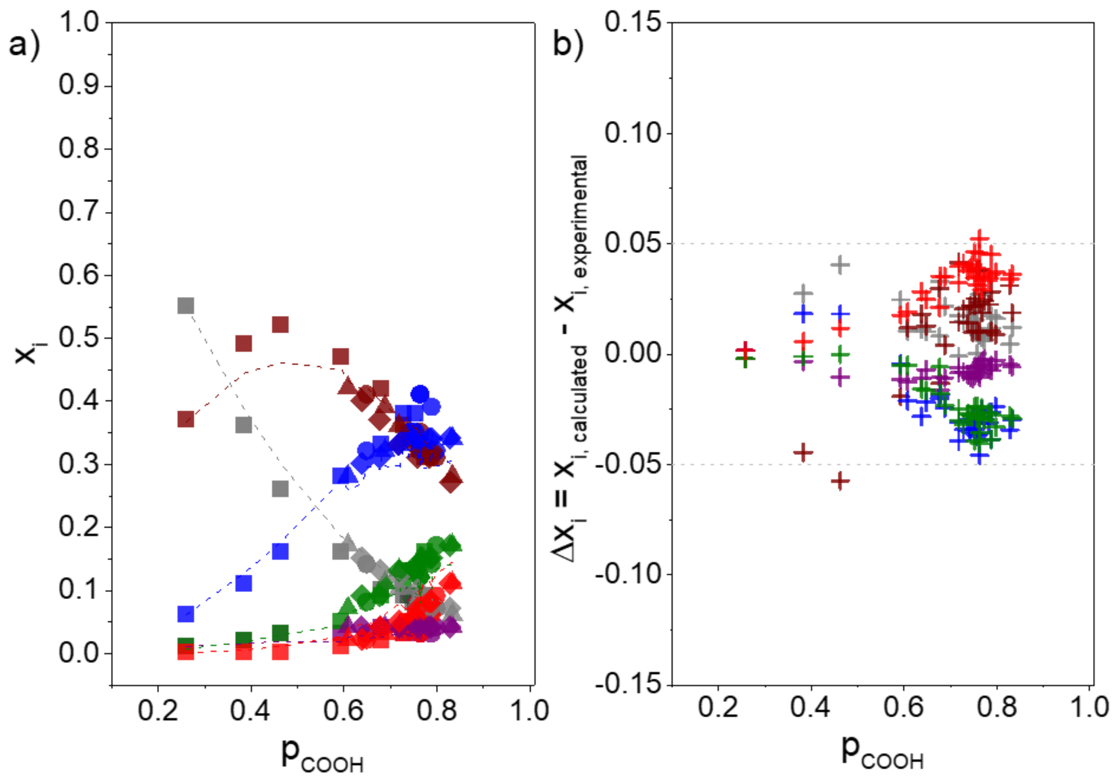

Figure S5: a) Molar fraction (x_i) of the residual glycerol (gray) and the repetitive units 1T (wine), 2T (purple), 1,3L (blue), 1,2L (green), and 1,2,3D (red), determined by ^1H NMR ($x_{i,\ experimental}$ - symbols), and calculated by **Equations 14 - 19** ($x_{i,\ calculated}$ - dashed lines), as a function of p_{COOH} (r = 0.67) for reactions performed using **CALB as catalyst in t-butanol** at 40 °C (■), 50 °C (●), 60 °C (▲), and 70 °C (◆).[5] **b)** Difference ($\Delta x_i = x_{i,\ calculated} - x_{i,\ experimental}$) between the molar fraction values determined experimentally by ^1H NMR and the calculated ones.

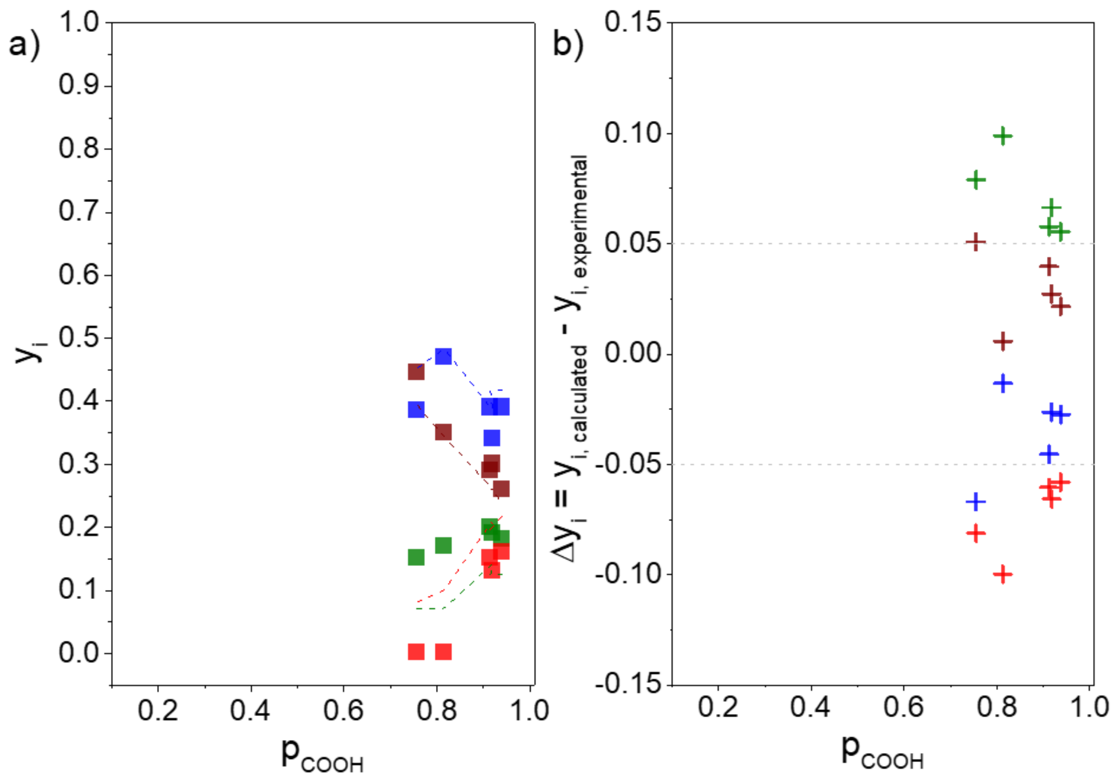

Figure S6: a) Molar fraction (y_i) of the repetitive units 1T (wine), 1,3L (blue), 1,2L (green), and 1,2,3D (red), determined by ^1H NMR ($y_{i,\ experimental}$ - symbols), and y_i calculated using **Equation S1** and x_i calculated using **Equations 14 - 19** ($y_{i,\ calculated}$ - dashed lines), as a function of p_{COOH} ($r = 0.67$) for reactions performed using **CALB as catalyst in bulk at 90 °C.**[1] **b)** Difference ($\Delta y_i = y_{i,\ calculated} - y_{i,\ experimental}$) between the molar fraction values determined experimentally by ^1H NMR and the calculated ones.

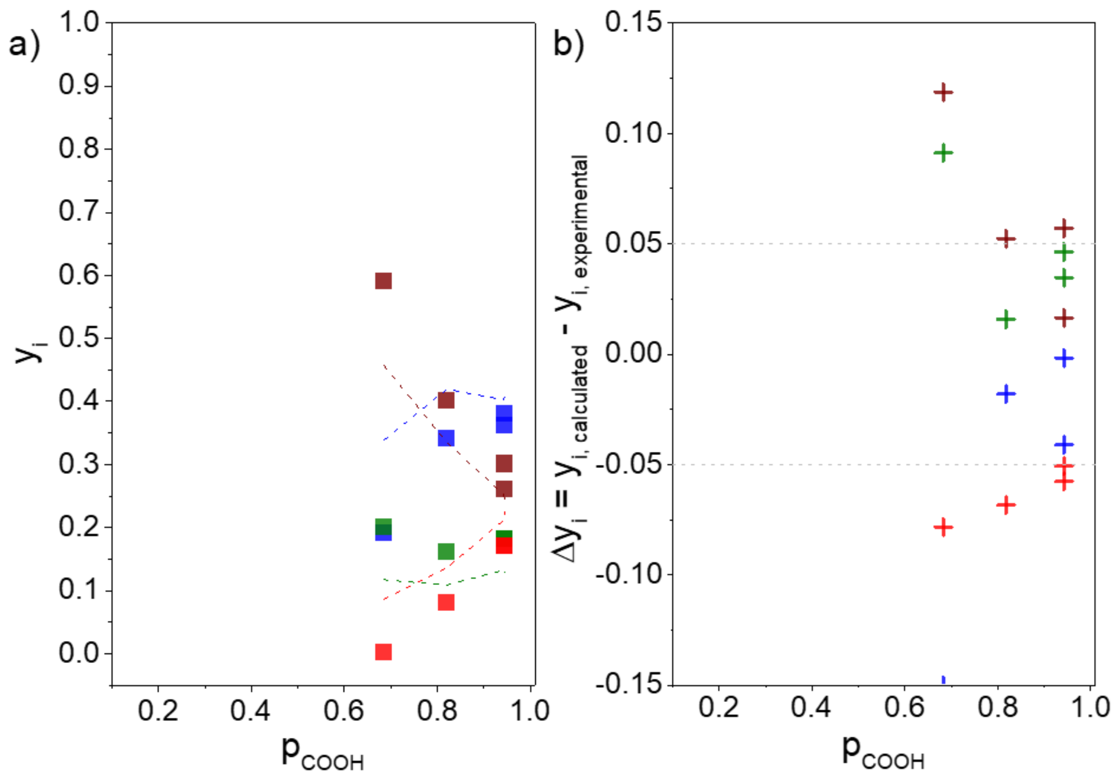

Figure S7: a) Molar fraction (y_i) of the repetitive units 1T (wine), 1,3L (blue), 1,2L (green), and 1,2,3D (red), determined by ^1H NMR ($y_{i,\ experimental}$ - symbols), and y_i calculated using **Equation S1** and x_i calculated using **Equations 14 - 19** ($y_{i,\ calculated}$ - dashed lines), as a function of p_{COOH} (r = 0.67) for reactions performed using **DBTO as catalyst in bulk at 150 °C.**[1] **b)** Difference ($\Delta y_i = y_{i,\ calculated} - y_{i,\ experimental}$) between the molar fraction values determined experimentally by ^1H NMR and the calculated ones.

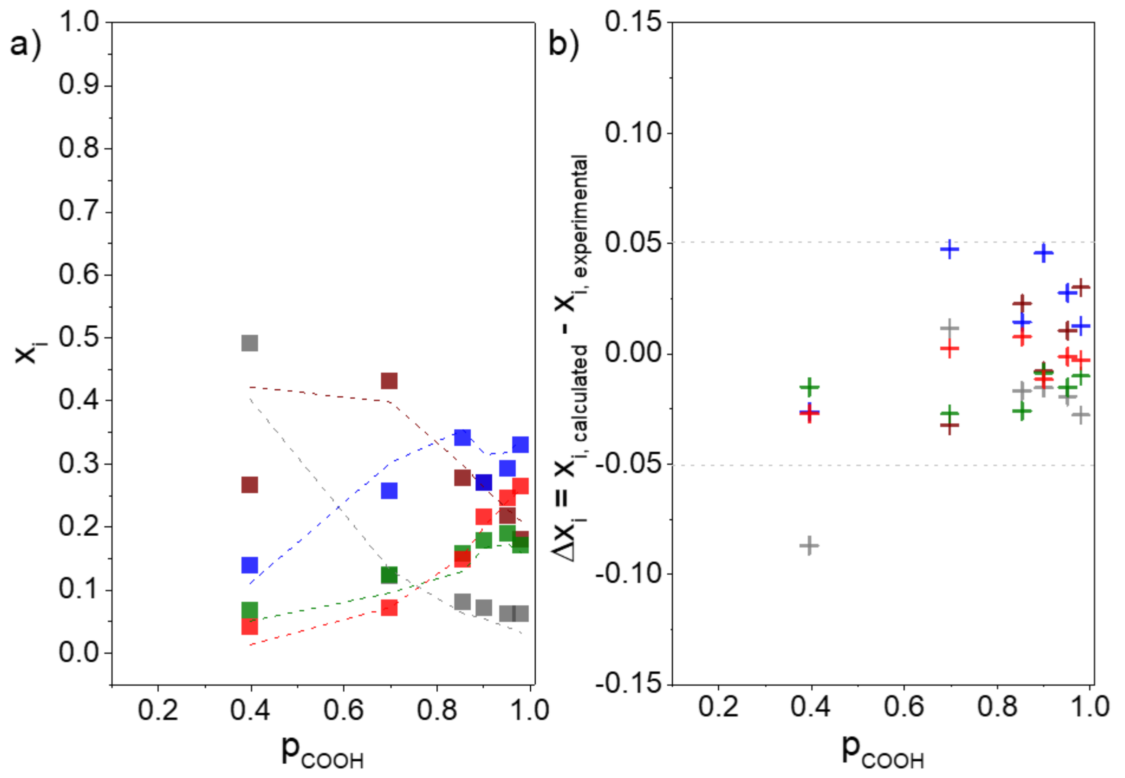

Figure S8: a) Molar fraction (x_i) of the residual glycerol (gray) and the repetitive units 1T (wine), 1,3L (blue), 1,2L (green), and 1,2,3D (red), determined by ^{1}H NMR ($x_{i,\, experimental}$ - symbols), and calculated by **Equations 14 - 19** ($x_{i,\, calculated}$ - dashed lines), as a function of p_{COOH} ($r = 0.67$) for reactions performed using **CALB as catalyst in diphenyl ether at 80 °C**.[3] **b)** Difference ($\Delta x_i = x_{i,\, calculated} - x_{i,\, experimental}$) between the molar fraction values determined experimentally by ^{1}H NMR and the calculated ones.

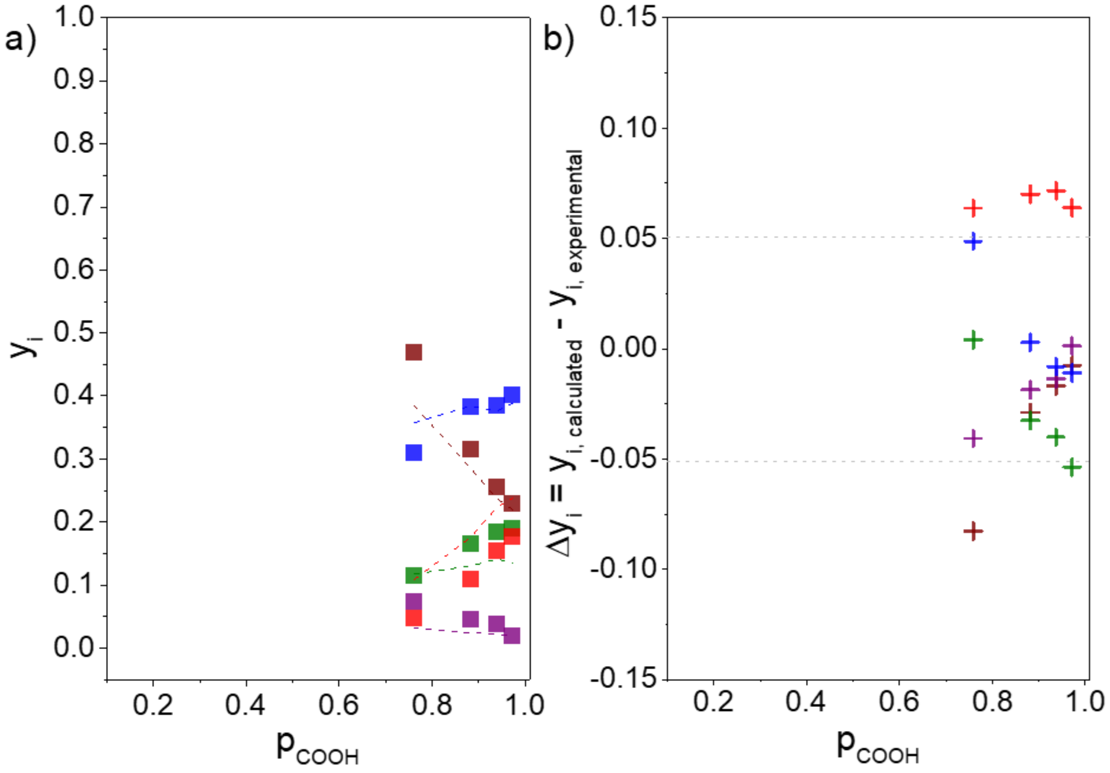

Figure S9: a) Molar fraction (y_i) of the repetitive units 1T (wine), 2T (purple), 1,3L (blue), 1,2L (green), and 1,2,3D (red), determined by ^{1}H NMR ($y_{i,\ experimental}$ - symbols), and y_i calculated using **Equation S1** and x_i calculated using **Equations 14 - 19** ($y_{i,\ calculated}$ - dashed lines), as a function of p_{COOH} ($r = 0.67$) for reactions performed **without catalyst in bulk at 150 °C.**[2] **b)** Difference ($\Delta y_i = y_{i,\ calculated} - y_{i,\ experimental}$) between the molar fraction values determined experimentally by ^{1}H NMR and the calculated ones.

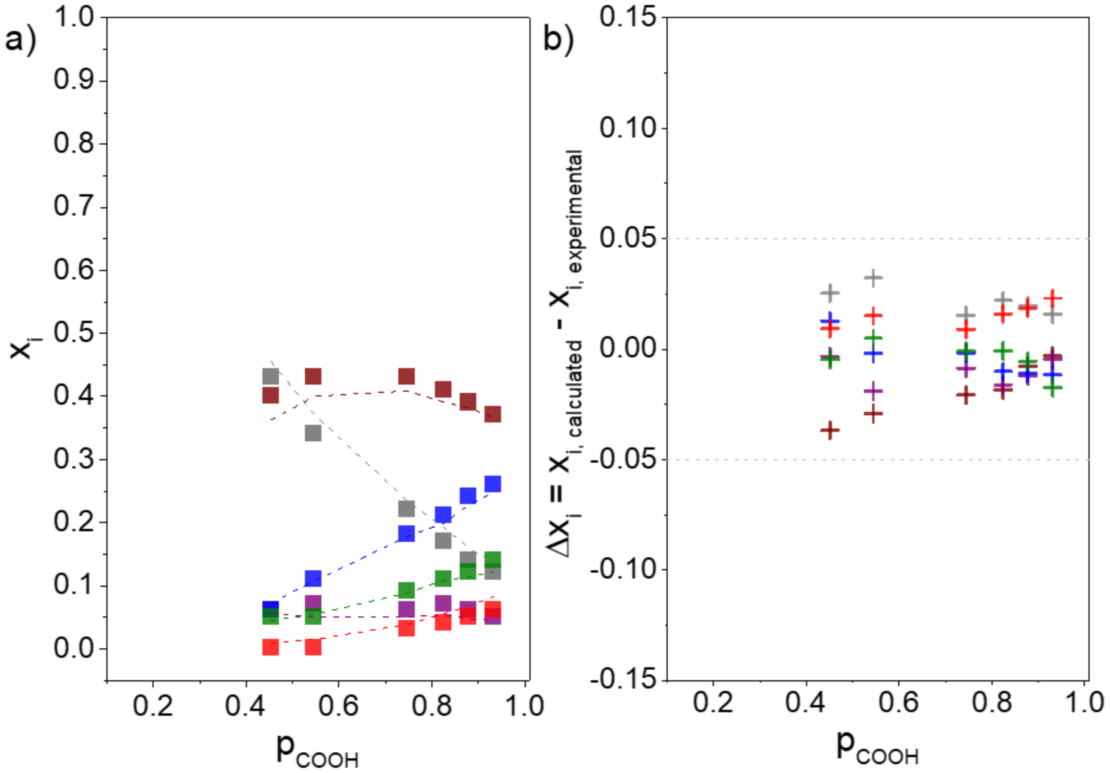

Figure S10: a) Molar fraction (x_i) of the residual glycerol (gray) and the repetitive units 1T (wine), 2T (purple), 1,3L (blue), 1,2L (green), and 1,2,3D (red), determined by ^{1}H NMR ($x_{i,\ experimental}$ - symbols), and calculated by **Equations 14 - 19** ($x_{i,\ calculated}$ - dashed lines), as a function of p_{COOH} (r = 0.50) for reactions performed using **DBTO as catalyst in bulk at 140 °C.**[4] **b)** Difference ($\Delta x_i = x_{i,\ calculated} - x_{i,\ experimental}$) between the molar fraction values determined experimentally by ^{1}H NMR and the calculated ones.

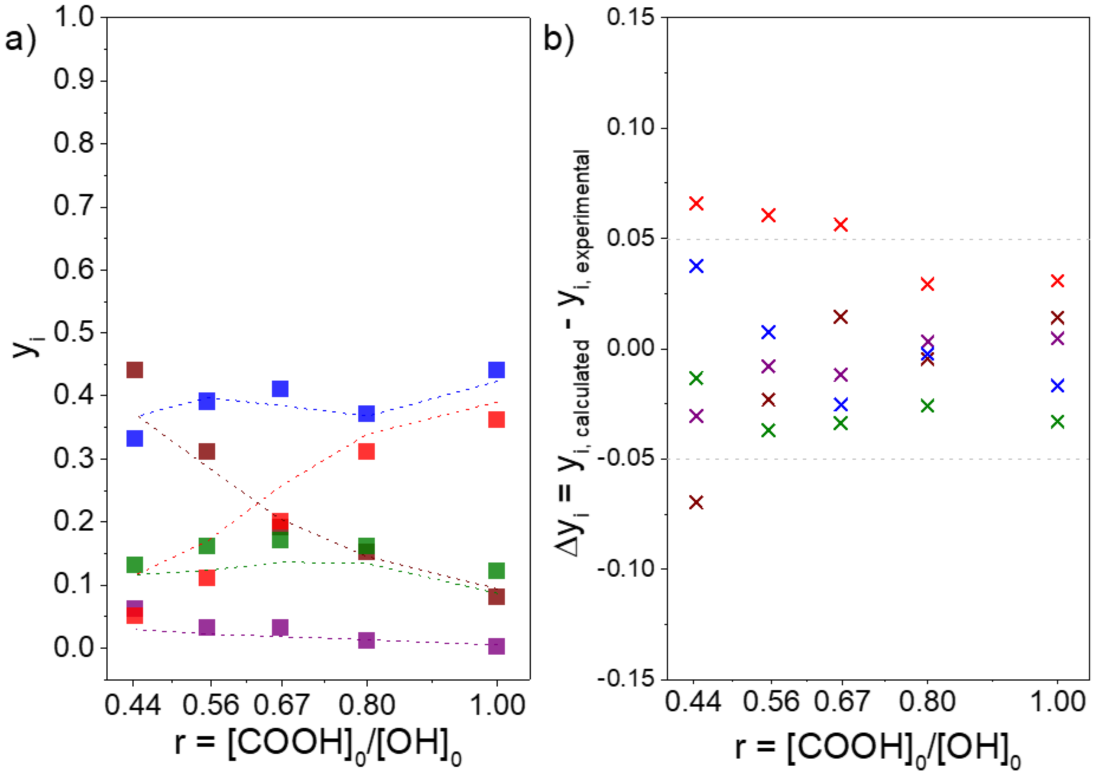

Figure S11: a) Molar fraction (y_i) of the repetitive units 1T (wine), 2T (purple), 1,3L (blue), 1,2L (green), and 1,2,3D (red), determined by ^{1}H NMR ($y_{i,\ experimental}$ - symbols), and y_i calculated using **Equation S1** and x_i calculated using **Equations 14 - 19** ($y_{i,\ calculated}$ - dashed lines), as a function of r = $[COOH]_0/[OH]_0$ for reactions performed **CALB as catalyst in acetone at 40 °C**.[5] **b)** Difference ($\Delta y_i = y_{i,\ calculated} - y_{i,\ experimental}$) between the molar fraction values determined experimentally by ^{1}H NMR and the calculated ones.

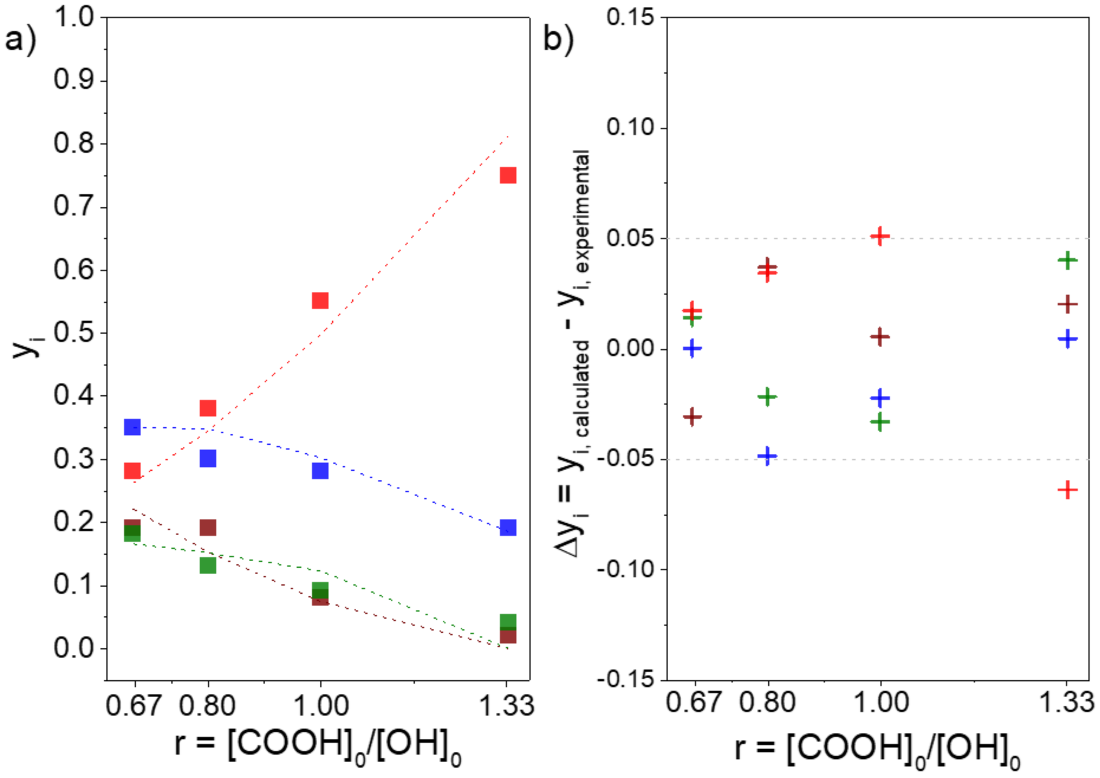

Figure S12: a) Molar fraction (y_i) of the repetitive units 1T (wine), 1,3L (blue), 1,2L (green), and 1,2,3D (red), determined by ^{1}H NMR ($y_{i,\ experimental}$ - symbols), and y_i calculated using **Equation S1** and x_i calculated using **Equations 14 - 19** ($y_{i,\ calculated}$ - dashed lines), as a function of $r = [COOH]_0/[OH]_0$ for reactions performed in using **CALB as catalyst diphenyl ether at 80 °C.**[3] **b)** Difference ($\Delta y_i = y_{i,\ calculated} - y_{i,\ experimental}$) between the molar fraction values determined experimentally by ^{1}H NMR and the calculated ones.

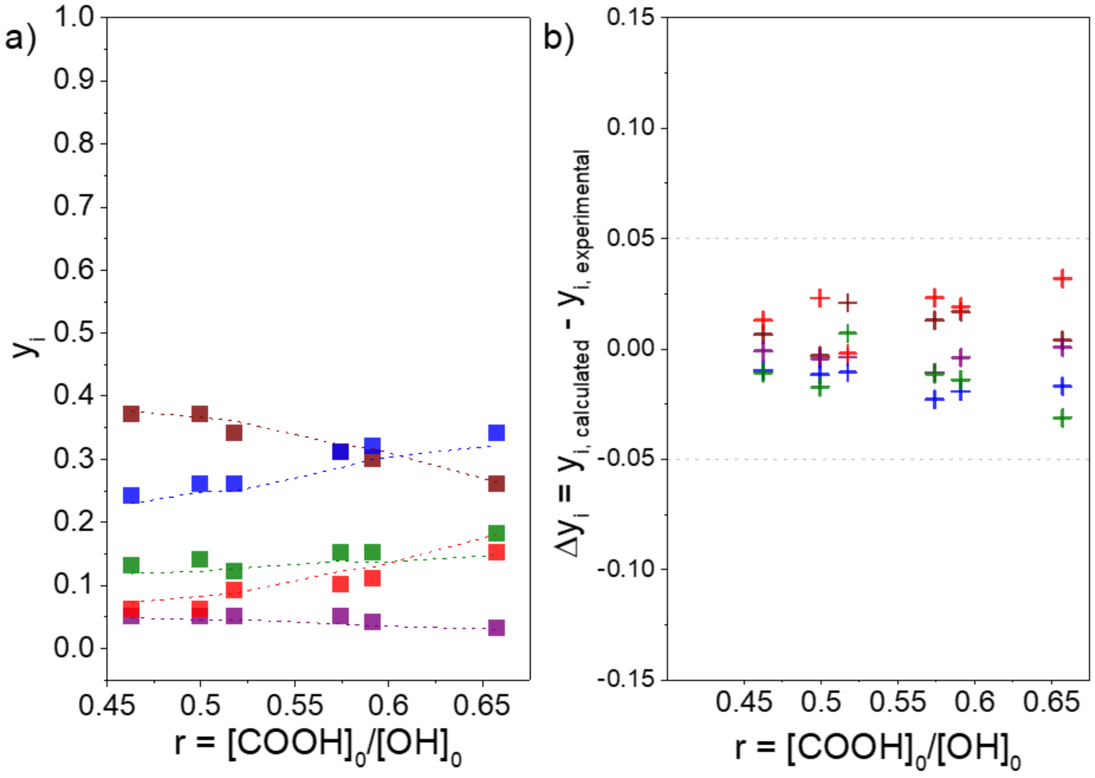

Figure S13: a) Molar fraction (y_i) of the repetitive units 1T (wine), 2T (purple), 1,3L (blue), 1,2L (green), and 1,2,3D (red), determined by ^{1}H NMR ($y_{i,\ experimental}$ - symbols), and y_i calculated using **Equation S1** and x_i calculated using **Equations 14 - 19** ($y_{i,\ calculated}$ - dashed lines), as a function of r = $[COOH]_0/[OH]_0$ for reactions performed in using **DBTO as catalyst bulk at 140 °C.**[4] **b)** Difference ($\Delta y_i = y_{i,\ calculated} - y_{i,\ experimental}$) between the molar fraction values determined experimentally by ^{1}H NMR and the calculated ones.

Table S6: Molar fraction (x_i) of the residual glycerol (G) and the repetitive units 1T, 2T, 1,3L, 1,2L, and 1,2,3D determined experimentally by ^{1}H NMR and calculated as reported by Li *et al.*[7], as well as the molar fractions calculated in the present work by using **Equation 14 - 19** and the (p_{OHp}/p_{OHs}) ratio as well as the difference ($\Delta x_i = x_{i,\ calculated} - x_{i,\ experimental}$) between the molar fraction values determined experimentally by ^{1}H NMR and the calculated ones.

	x_G	x_{1T}	x_{2T}	$x_{1,3L}$	$x_{1,2L}$	$x_{1,2,3D}$	DB (%)
^{1}H NMR Data[7]	0.00	0.15	0.00	0.47	0.10	0.28	50
Li *et al.*[7]	0.06	0.19	0.10	0.47	0.29	0.22	37
Present work	0.01	0.14	0.01	0.47	0.08	0.29	51
Δx_i (Li *et al.*[7])	0.06	0.04	0.10	0.00	0.19	0.06	-13
Δx_i (Present work)	0.01	-0.01	0.01	0.00	-0.02	0.01	1

Data for Figure 7

Figure 7 shows a “Theoretical Structural Map” for polyesters based on glycerol, a plot of the ($p_{OH,p}$/$p_{OH,s}$) ratio as a function of p_{OH} for variable degree of branching (DB – calculated using **Equation 22**, **Figure 7a**) as well as Dp_n (**Equation 23**) as a function of p_{OH} for variable ($p_{OH,p}$/$p_{OH,s}$) ratios (**Figure 7b**). **Figure 7a** and **Figure 7b** were constructed using the data calculated using **Spreadsheet S1** and available in **Table S7** and **Tables S8 – S11**, respectively.

Table S7: Conversion of hydroxy groups (p_{OH}) for a given degree of branching (DB) at variable ($p_{OH,p}$/$p_{OH,s}$) ratios determined using **Spreadsheet S1**. These data were used for the construction of **Figure 7a**. The color of the numbers and the cells corresponds to the colors of the curves and the pink area in **Figure 7a**, respectively.

DB = 10%		DB = 20%		DB = 30%		DB = 40%		DB = 50%		DB = 60%		DB = 70%		DB = 80%		DB = 90%		DB = 100%	
p_{OH}	$\frac{p_{OH,p}}{p_{OH,s}}$	p_{OH}	$\frac{p_{OH,p}}{p_{OH,s}}$	p_{OH}	$\frac{p_{OH,p}}{p_{OH,s}}$	p_{OH}	$\frac{p_{OH,p}}{p_{OH,s}}$	p_{OH}	$\frac{p_{OH,p}}{p_{OH,s}}$	p_{OH}	$\frac{p_{OH,p}}{p_{OH,s}}$	p_{OH}	$\frac{p_{OH,p}}{p_{OH,s}}$	p_{OH}	$\frac{p_{OH,p}}{p_{OH,s}}$	p_{OH}	$\frac{p_{OH,p}}{p_{OH,s}}$	p_{OH}	$\frac{p_{OH,p}}{p_{OH,s}}$
0.14	1	0.27	1	0.39	1	0.50	1	0.60	1	0.69	1	0.78	1	0.86	1	0.93	1	0.999	1
0.16	2	0.30	2	0.43	2	0.55	2	0.67	2	0.77	2	0.84	1.8	0.89	1.5	0.94	1.2	0.999	1.1
0.19	3	0.35	3	0.50	3	0.65	3	0.78	3	0.78	2.1	---	---	---	---	---	---	---	---
0.21	4	0.40	4	0.58	4	0.75	4	---	---	---	---	---	---	---	---	---	---	---	---
0.24	5	0.46	5	0.66	5	---	---	---	---	---	---	---	---	---	---	---	---	---	---
0.27	6	0.52	6	0.72	5.5	---	---	---	---	---	---	---	---	---	---	---	---	---	---
0.30	7	0.58	7	---	---	---	---	---	---	---	---	---	---	---	---	---	---	---	---
0.33	8	0.63	8	---	---	---	---	---	---	---	---	---	---	---	---	---	---	---	---
0.36	9	0.69	9	---	---	---	---	---	---	---	---	---	---	---	---	---	---	---	---
0.39	10	0.70	9.2	---	---	---	---	---	---	---	---	---	---	---	---	---	---	---	---
0.42	11	---	---	---	---	---	---	---	---	---	---	---	---	---	---	---	---	---	---
0.45	12	---	---	---	---	---	---	---	---	---	---	---	---	---	---	---	---	---	---
0.48	13	---	---	---	---	---	---	---	---	---	---	---	---	---	---	---	---	---	---
0.51	14	---	---	---	---	---	---	---	---	---	---	---	---	---	---	---	---	---	---
0.54	15	---	---	---	---	---	---	---	---	---	---	---	---	---	---	---	---	---	---
0.57	16	---	---	---	---	---	---	---	---	---	---	---	---	---	---	---	---	---	---
0.60	17	---	---	---	---	---	---	---	---	---	---	---	---	---	---	---	---	---	---
0.63	18	---	---	---	---	---	---	---	---	---	---	---	---	---	---	---	---	---	---
0.67	19	---	---	---	---	---	---	---	---	---	---	---	---	---	---	---	---	---	---
0.68	20	---	---	---	---	---	---	---	---	---	---	---	---	---	---	---	---	---	---

Table S8: Average degree of polymerization (Dp_n) as a function of the conversion of hydroxy groups (p_{OH}) for variable ($p_{OH,p}$/$p_{OH,s}$) ratios and at molar ratio **r = 0.5**, calculated using **Spreadsheet S1** and used for the construction of **Figure 7b**. The color of the table columns corresponds to one of the curves in **Figure 7b**.

**r = 0.5**									
**$p_{OH,p}$/$p_{OH,s}$ = 1**		**$p_{OH,p}$/$p_{OH,s}$ = 2**		**$p_{OH,p}$/$p_{OH,s}$ = 5**		**$p_{OH,p}$/$p_{OH,s}$ = 10**		**$p_{OH,p}$/$p_{OH,s}$ = 20**	
**p_{OH}**	**Dp_n**	**p_{OH}**	**Dp_n**	**p_{OH}**	**Dp_n**	**p_{OH}**	**Dp_n**	**p_{OH}**	**Dp_n**
0.025	1.0	0.025	1.0	0.025	1.0	0.025	1.0	0.025	1.0
0.050	1.1	0.050	1.1	0.050	1.1	0.050	1.1	0.050	1.1
0.100	1.2	0.100	1.2	0.100	1.2	0.100	1.2	0.100	1.2
0.150	1.3	0.150	1.3	0.150	1.3	0.150	1.3	0.150	1.3
0.200	1.5	0.200	1.5	0.200	1.5	0.200	1.5	0.200	1.5
0.250	1.7	0.250	1.7	0.250	1.7	0.250	1.7	0.250	1.7
0.300	2.0	0.300	2.0	0.300	2.0	0.300	2.0	0.300	2.0
0.350	2.6	0.350	2.6	0.350	2.5	0.350	2.5	0.350	2.5
0.375	3.0	0.375	2.9	0.375	2.9	0.375	2.8	0.375	2.8
0.400	3.5	0.400	3.5	0.400	3.4	0.400	3.3	0.400	3.3
0.425	4.4	0.425	4.3	0.425	4.1	0.425	4.1	0.425	4.0
0.450	5.8	0.450	5.6	0.450	5.3	0.450	5.2	0.450	5.1
0.475	8.6	0.475	8.2	0.475	7.6	0.475	7.3	0.475	7.2
0.488	11.4	0.488	10.7	0.488	9.6	0.488	9.2	0.488	9.0
0.495	14.2	0.495	13.1	0.495	11.5	0.495	10.9	0.495	10.6
0.500	17.0	0.500	15.5	0.500	13.3	0.500	12.5	0.500	12.1

Table S9: Average degree of polymerization (Dp_n) as a function of the conversion of hydroxy groups (p_{OH}) for a variable ($p_{OH,p}/p_{OH,s}$) ratio and at molar ratio **r = 0.67**, calculated using **Spreadsheet S1** and used for the construction of **Figure 7b**. The color of the table columns corresponds to one of the curves in **Figure 7b**.

**r = 0.67**									
**$p_{OH,p}/p_{OH,s}$ = 1**		**$p_{OH,p}/p_{OH,s}$ = 2**		**$p_{OH,p}/p_{OH,s}$ = 5**		**$p_{OH,p}/p_{OH,s}$ = 10**		**$p_{OH,p}/p_{OH,s}$ = 20**	
**p_{OH}**	**Dp_n**	**p_{OH}**	**Dp_n**	**p_{OH}**	**Dp_n**	**p_{OH}**	**Dp_n**	**p_{OH}**	**Dp_n**
0.033	1.0	0.033	1.0	0.033	1.0	0.033	1.0	0.033	1.0
0.067	1.1	0.067	1.1	0.067	1.1	0.067	1.1	0.067	1.1
0.133	1.2	0.133	1.2	0.133	1.2	0.133	1.2	0.133	1.2
0.200	1.3	0.200	1.3	0.200	1.3	0.200	1.3	0.200	1.3
0.267	1.4	0.267	1.4	0.267	1.4	0.267	1.4	0.267	1.4
0.333	1.6	0.333	1.6	0.333	1.6	0.333	1.6	0.333	1.6
0.400	2.0	0.400	2.0	0.400	1.9	0.400	1.9	0.400	1.9
0.467	2.6	0.467	2.5	0.467	2.5	0.467	2.4	0.467	2.4
0.500	3.1	0.500	3.0	0.500	2.9	0.500	2.9	0.500	2.8
0.533	3.8	0.533	3.7	0.533	3.6	0.533	3.5	0.533	3.4
0.567	5.2	0.567	5.0	0.567	4.6	0.567	4.5	0.567	4.5
0.613	10.4	0.617	10.4	0.600	6.9	0.600	6.6	0.600	6.5
0.638	25.0	0.642	25.0	0.633	13.8	0.633	13.0	0.633	12.7
0.647	48.8	0.652	55.0	0.650	29.0	0.650	26.0	0.650	25.0
0.652	109.4	0.655	101.8	0.657	52.6	0.658	51.4	0.659	52.3
--	--	--	--	0.661	103.8	0.662	101.2	0.663	106.3

Table S10: Average degree of polymerization (Dp_n) as a function of the conversion of hydroxy groups (p_{OH}) for a variable ($p_{OH,p}/p_{OH,s}$) ratio and at molar ratio **r = 0.83**, calculated using **Spreadsheet S1** and used for the construction of **Figure 7b**. The color of the table columns corresponds to one of the curves in **Figure 7b**.

**r = 0.83**									
$p_{OH,p}/p_{OH,s}$ = 1		$p_{OH,p}/p_{OH,s}$ = 2		$p_{OH,p}/p_{OH,s}$ = 5		$p_{OH,p}/p_{OH,s}$ = 10		$p_{OH,p}/p_{OH,s}$ = 20	
p_{OH}	Dp_n	p_{OH}	Dp_n	p_{OH}	Dp_n	p_{OH}	Dp_n	p_{OH}	Dp_n
0.042	1.0	0.042	1.0	0.042	1.0	0.042	1.0	0.042	1.0
0.083	1.1	0.083	1.1	0.083	1.1	0.083	1.1	0.083	1.1
0.167	1.1	0.167	1.1	0.167	1.1	0.167	1.1	0.167	1.1
0.250	1.2	0.250	1.2	0.250	1.2	0.250	1.2	0.250	1.2
0.333	1.4	0.333	1.4	0.333	1.4	0.333	1.4	0.333	1.4
0.417	1.6	0.417	1.6	0.417	1.6	0.417	1.6	0.417	1.6
0.500	2.0	0.500	2.0	0.500	2.0	0.500	1.9	0.500	1.9
0.542	2.4	0.542	2.3	0.542	2.3	0.583	2.7	0.583	2.6
0.583	2.9	0.583	2.8	0.583	2.7	0.625	3.4	0.625	3.4
0.625	3.9	0.625	3.7	0.625	3.5	0.667	5.0	0.667	5.0
0.667	6.0	0.667	5.6	0.667	5.1	0.700	8.6	0.683	6.3
0.708	14.2	0.708	12.2	0.708	10.7	--	--	--	--
0.721	25.2	0.725	24.5	0.734	38.1	--	--	--	--
0.729	53.1	0.733	50.8	--	--	--	--	--	--
0.733	107.1	0.738	111.5	--	--	--	--	--	--

Table S11: Average degree of polymerization (Dp_n) as a function of the conversion of hydroxy groups (p_{OH}) for a variable ($p_{OH,p}/p_{OH,s}$) ratio and at molar ratio **r = 1.00**, calculated using **Spreadsheet S1** and used for the construction of **Figure 7b**. The color of the table columns corresponds to one of the curves in **Figure 7b**.

r = 1.00									
$p_{OH,p}/p_{OH,s}$ = 1		$p_{OH,p}/p_{OH,s}$ = 2		$p_{OH,p}/p_{OH,s}$ = 5		$p_{OH,p}/p_{OH,s}$ = 10		$p_{OH,p}/p_{OH,s}$ = 20	
p_{OH}	Dp_n	p_{OH}	Dp_n	p_{OH}	Dp_n	p_{OH}	Dp_n	p_{OH}	Dp_n
0.050	1.0	0.050	1.0	0.050	1.0	0.050	1.0	0.050	1.0
0.100	1.0	0.100	1.0	0.100	1.0	0.100	1.0	0.100	1.0
0.200	1.1	0.200	1.1	0.200	1.1	0.200	1.1	0.200	1.1
0.300	1.2	0.300	1.2	0.300	1.2	0.300	1.2	0.300	1.2
0.400	1.4	0.400	1.4	0.400	1.4	0.400	1.3	0.400	1.3
0.500	1.7	0.500	1.6	0.500	1.6	0.500	1.6	0.500	1.6
0.600	2.3	0.600	2.2	0.600	2.2	0.600	2.1	0.600	2.1
0.650	3.0	0.650	2.9	0.650	2.7	0.650	2.7	0.650	2.7
0.700	4.7	0.700	4.4	0.700	4.1	0.675	3.2	0.675	3.2
0.750	13.0	0.750	10.9	0.734	6.7	0.700	4.1	0.683	3.4
0.765	31.3	0.765	21.4	--	--	--	--	--	--
0.769	47.3	0.770	32.2	--	--	--	--	--	--
0.770	60.7	0.774	54.4	--	--	--	--	--	--
0.772	84.7	0.776	83.6	--	--	--	--	--	--
0.772	104.1	0.777	102.9	--	--	--	--	--	--

References

(1) Yang, Y.; Lu, W.; Cai, J.; Hou, Y.; Ouyang, S.; Xie, W.; Gross, R. A. Poly(Oleic Diacid-co-Glycerol): Comparison of Polymer Structure Resulting from Chemical and Lipase Catalysis. *Macromolecules* **2011**, *44*, 1977–1985. https://doi.org/10.1021/bk-2012-1105.ch008.

(2) Pin, J. M.; Valerio, O.; Misra, M.; Mohanty, A. Impact of Butyl Glycidyl Ether Comonomer on Poly(Glycerol-Succinate) Architecture and Dynamics for Multifunctional Hyperbranched Polymer Design. *Macromolecules* **2017**, *50* (3), 732–745. https://doi.org/10.1021/acs.macromol.6b02424.

(3) Rao, Z. K.; Ni, H. L.; Li, Y.; Zhu, H. Y.; Liu, Y.; Hao, J. Y. Macroscopic Scaffold Control for Lipase-Catalyzed Dendritic Polyol-Polyesters. *Macromol. Chem. Phys.* **2019**, *220* (11), 1–10. https://doi.org/10.1002/macp.201900048.

(4) Zhang, T.; Howell, B. A.; Dumitrascu, A.; Martin, S. J.; Smith, P. B. Synthesis and Characterization of Glycerol-Adipic Acid Hyperbranched Polyesters. *Polym. (United Kingdom)* **2014**, *55* (20), 5065–5072. https://doi.org/10.1016/j.polymer.2014.08.036.

(5) Perin, G. B.; Felisberti, M. I. Enzymatic Synthesis of Poly(Glycerol Sebacate): Kinetics, Chain Growth, and Branching Behavior. *Macromolecules* **2020**, *53* (18), 7925–7935. https://doi.org/10.1021/acs.macromol.0c01709.

(6) Perin, G. B.; Felisberti, M. I. Mechanism and Kinetics of Lipase-Catalyzed Polycondensation of Glycerol and Sebacic Acid: Influence of Solvent and Temperature. *Biomacromolecules* **2022**, *23* (7), 2968–2975. https://doi.org/10.1021/acs.biomac.2c00458.

(7) Li, Y.; Cook, W. D.; Moorhoff, C.; Huang, W.; Chen, Q. Synthesis, Characterization and Properties of Biocompatible Poly(Glycerol Sebacate) Pre-Polymer and Gel. *Polym. Int.* **2013**, *62*, 534–547. https://doi.org/10.1002/pi.4419.
